# Putting the Pieces Together in Gilles de la Tourette Syndrome: Exploring the Link Between Clinical Observations and the Biological Basis of Dysfunction

**DOI:** 10.1007/s10548-016-0525-z

**Published:** 2016-10-25

**Authors:** Rowshanak Hashemiyoon, Jens Kuhn, Veerle Visser-Vandewalle

**Affiliations:** 1 Department of Stereotactic and Functional Neurosurgery, University Hospital of Cologne, Kerpener Strasse 62, 50937 Cologne, Germany; 2 Department of Psychiatry and Psychotherapy, University Hospital of Cologne, Cologne, Germany; 3 Johanniter Hospital, EVKLN, Oberhausen, Germany

**Keywords:** Basal ganglia, Tic, Oscillation, Premonitory urge, Centre médian/parafascicular nucleus of the thalamus (CM/Pf), Deep brain stimulation, Gamma, Theta/gamma cross frequency coupling, OCD, ADHD, Movement Disorder, Genetics

## Abstract

Gilles de la Tourette syndrome is a complex, idiopathic neuropsychiatric disorder whose pathophysiological mechanisms have yet to be elucidated. It is phenotypically heterogeneous and manifests more often than not with both motor and behavioral impairment, although tics are its clinical hallmark. Tics themselves present with a complex profile as they characteristically wax and wane and are often preceded by premonitory somatosensory sensations to which it is said a tic is the response. Highly comorbid with obsessive–compulsive disorder and attention deficit-hyperactivity disorder, it is purported to be an epigenetic, neurodevelopmental spectrum disorder with a complex genetic profile. It has a childhood onset, occurs disproportionately in males, and shows spontaneous symptomatic attenuation by adulthood in the majority of those afflicted. Although not fully understood, its neurobiological basis is linked to dysfunction in the cortico-basal ganglia–thalamo–cortical network. Treatment modalities for Tourette syndrome include behavioral, pharmacological and surgical interventions, but there is presently no cure for the disorder. For those severely affected, deep brain stimulation (DBS) has recently become a viable therapeutic option. A key factor to attaining optimal results from this surgery is target selection, a topic still under debate due to the complex clinical profile presented by GTS patients. Depending on its phenotypic expression and the most problematic aspect of the disorder for the individual, one of three brain regions is most commonly chosen for stimulation: the thalamus, globus pallidus, or nucleus accumbens. Neurophysiological analyses of intra- and post-operative human electrophysiological recordings from clinical DBS studies suggest a link between tic behavior and activity in both the thalamus and globus pallidus. In particular, chronic recordings from the thalamus have shown a correlation between symptomatology and (1) spectral activity in gamma band power and (2) theta/gamma cross frequency coherence. These results suggest gamma oscillations and theta/gamma cross correlation dynamics may serve as biomarkers for dysfunction. While acute and chronic recordings from human subjects undergoing DBS have provided better insight into tic genesis and the neuropathophysiological mechanisms underlying Tourette syndrome, these studies are still sparse and the field would greatly benefit from further investigations. This review reports data and discoveries of scientific and clinical relevance from a wide variety of methods and provides up-to-date information about our current understanding of the pathomechanisms underlying Tourette syndrome. It gives a comprehensive overview of the current state of knowledge and addresses open questions in the field.

## Introduction

Gilles de la Tourette syndrome (GTS) is a complex neurological disorder of unknown etiology affecting approximately 1 % of the population (Robertson 2008; American Psychiatric Association 2013). Although it is most readily associated with sudden outbursts of socially inappropriate locutions, it is a neuropsychiatric disorder consisting of considerable motor as well as behavioral impairment. Diagnosis is determined by the childhood onset of multiple motor tics and at least one phonic tic (not necessarily present concurrently), which collectively must persist for at least one cumulative year and are not precipitated by known medical causes or pharmacological agents (Robertson 2000; American Psychiatric Association 2013).

Symptoms typically occur at a mean onset age of about 4–7 years old, peak in severity prepubertally at about 10–12 years old, and remit or become subclinical in two-thirds to three-fourths of the patient population by adulthood (Freeman et al. 2000; Leckman 2002; Bloch and Leckman 2009; Leckman et al. 2010; Felling and Singer 2011). Consistently, GTS has a reported four to five fold higher incidence in children than adults (Robertson 2000; Burd et al. 2005; Hariz and Robertson 2010). This decline in symptomatology during maturation supports the theory that GTS is a neurodevelopmental disorder in which certain brain structures and/or processes must yet mature for proper brain function (Kurlan et al. 2002; Ghanizadeh and Mosallaei 2009).

At approximately 10 years of age premonitory urges are typically expressed, coincident with first observations of tic suppression (Cohen and Leckman 1992; Kwak et al. 2003; Leckman et al. 2010; McNaught and Mink 2011). These sensations—which are frequently more distressful than the tics themselves—are anatomically localized to the region associated with the impending tic and precede the need for action; thus, they appear to signal the oncoming tic, making its suppression possible (Cohen and Leckman 1992; Leckman et al. 1993). Tic expression in GTS has been compared to sneezing: one feels the sensation coming on, could suppress it if necessary, but feels relief once expressed. The caveat here is that suppression of the tic is (1) transitory and (2) often results in an increased rebound severity of expression post-suppression (Leckman 2003; Grados and Mathews 2009). As they are unique to tic disorders and Tourette syndrome, the presence of premonitory sensations can be used to distinguish GTS from other movement disorders (McNaught and Mink 2011).

Tics are sudden, repetitive and purposeless and can be either motor or phonic^1^ and classified as either simple or complex. Simple tics, whether brief and jerk-like or slow and sustained, appear meaningless, while complex tics involve the coordination of multiple muscle groups and may or may not appear purposeful (Jankovic and Stone 1991). Simple motor tics involve one muscle group and can be tonic (>500 ms), dystonic (>300 ms), and/or clonic (<100 ms) (Jankovic 1997). Simple tonic tics are exemplified by isometric contractions such as tensing of the abdominal muscles, dystonic tics include shoulder rotation and oculogyric deviation, and clonic tics are rapid movements such as eye blinking, facial twitching, and head, neck, or limb jerking (Jankovic and Stone 1991; Jankovic 2001). Simple phonic tics commonly include throat clearing, coughing, or grunting. In contrast to simple tics, complex tics involve several muscle groups and can appear either intentional or unintentional. They consist of coordinated movements such as gesturing, hopping, and body jerking. Phonically, they may be expressed as humming, animal sounds, and coprolalia (the uncontrollable outburst of obscenities or socially inappropriate utterances).

Interestingly, although coprolalia is the behavior most readily associated with Tourette syndrome, it is both not specific to it and not present in the majority of patients. Time of onset for this symptom has been reported at 2.2, ~4, and 5 years after tic onset and usually presents by age fifteen (Goldenberg et al. 1994; Robertson 2000; Freeman et al. 2009; Fahn et al. 2011; McNaught and Mink 2011). A longitudinal study, which followed 61 patients from first onset of symptoms to most recent medical visit in those who had a minimum of 4 years (and an average of 8 years) between symptom expression and physician visit, reported 9.8 % exhibited coprolalia. Nonetheless, although typically cited at 10 %, prevalence estimates vary from 4 % to greater than 30 % of the patient population, with discrepancies attributed to cultural differences; disease severity; gender; and age incongruities. As with other symptoms of GTS, this one increases and then declines with age, peaking in adolescence (Goetz et al. 1992; Goldenberg et al. 1994).

An overwhelming majority of GTS patients present with comorbidities, which are most commonly comprised of attention deficit-hyperactivity disorder (ADHD) and obsessive–compulsive disorder (OCD) (at a frequency of up to 80 % each), but also include anxiety (49 %), learning disabilities (47 %), depression (25 %), and behavioral problems (26 %), as well as self-injurious behavior and reduced executive function, among others (Baron-Cohen et al. 1999; Coffey et al. 2000; Robertson 2000; Kurlan et al. 2002; Burd et al. 2005; Robertson and Orth 2006; Freeman 2007; Roessner et al. 2007; Ghanizadeh and Mosallaei 2009; Hariz and Robertson 2010). Symptom severity is reported to be positively correlated with comorbidity rates (Melillo and Leisman 2009). 

GTS has been genetically linked with OCD and ADHD (suggesting that they are part of a spectrum), although their profiles are notably dissimilar (Barkley 1993; Albin and Mink 2006). The incidence of “pure” or “uncomplicated” GTS is uncommon, present in only about 10 % of the observed GTS population (Robertson 2000; Faraone et al. 2003; Mohammadi et al. 2004; Pittenger et al. 2011; Eapen and Robertson 2015; Ganos and Martino 2015). In contrast, both OCD and ADHD present with low comorbidity rates, but show a higher prevalence in the general population. Despite the very high comorbidity of GTS with OCD and ADHD, the reverse comorbidity of OCD and ADHD with GTS is typically reported to be only about 2–7 % (Zohar et al. 1992; Barkley 1993; Denys et al. 2004).

Based on clinical characteristics and comorbidities, GTS may be subtyped into three categories: (1) “pure” GTS, comprised primarily of motor and phonic tics; (2) “full blown” GTS, which included copro-, echo-, and paliphenomena; and (3) GTS+, which includes OCD, ADHD, and other severe psychopathologies such as self-injurious behavior, depression, personality disorders, etc.… (Robertson 2000; Grados and Mathews 2009; Ganos and Martino 2015). Comorbidities may provide the underlying structure for the broad range of phenotypic expression, and as such, are a key variable to explicating the complexity of the clinical profile. Largely, pure GTS does not typically present with severe motor or executive dysfunction, although these individuals are suggested to exhibit more obsessive–compulsive and self-injurious behavior and are more “delinquent” than GTS + ADHD, likely as a result of rage or anger (Robertson 2000; Rizzo et al. 2007; Cavanna et al. 2008). GTS comorbid with ADHD presents with appreciable cognitive and behavioral disability, and GTS co-occurring with both ADHD and OCD presents as more heritable with motor tics appearing at an earlier age (Grados and Mathews 2008, 2009). More often than not, it is the disability resulting from comorbid symptomatology rather than GTS itself which prompts a patient to seek medical attention (Robertson 2000).

The predominance of comorbidities together with variations in tic phenomenology suggests dysfunction of multiple neurotransmitter systems (Buse et al. 2013). Although the neurobiological basis of GTS is unclear, the effectiveness of dopamine antagonists in relieving tics has given rise to multiple hypotheses for its role in GTS (Buse et al. 2013). Further imaging, physiological and pathological studies have indicated a pivotal role for the re-entrant circuits of the cortico-basal ganglia–thalamo–cortical network, thus implicating gamma-amino-butyric acid (GABA) and glutamate as well (Felling and Singer 2011; Draper et al. 2014), and suggesting the underlying problem may be one of action selection (Mink 2001; Albin and Mink 2006). Despite significant strides in the field, neither the underlying neuropathological mechanism(s), nor the primary initiation site(s) of dysfunction are fully elucidated.

GTS may be managed pharmacologically, behaviorally, or surgically, all of which aim at ameliorating symptoms. Preferred pharmaceutical therapies are comprised of antipsychotics or, in milder cases, amphetamines (McNaught and Mink 2011). And while comprehensive behavioral intervention therapy has shown good success, few trained professionals are available as of yet (Piacentini et al. 2010; Capriotti et al. 2014). Severely afflicted patients tend to be refractory to both behavioral and pharmacological therapies and thus may opt for surgical intervention (Porta et al. 2009a; Hariz and Robertson 2010; Sassi et al. 2011).

Historically, medically-refractory patients were treated by neurosurgical ablation in a variety of targets with diverse results (Hassler and Dieckmann 1970; Temel and Visser-Vandewalle 2004; Singer 2010). Despite delivering symptomatic relief in many cases, outcomes include complications of mild to severe permanent deficits as well. In 1999, however, deep brain stimulation (DBS) was introduced as a possible alternative treatment (Vandewalle et al. 1999). Since then, over 120 patients worldwide have been reported to have undergone the procedure with varying degrees of success (Sassi et al. 2011; Schrock et al. 2014). While the use of DBS is a relatively new remedy available for treatment-refractory GTS, successful outcomes have been reported in many cases (Maling et al. 2012).

A key factor to attaining optimal results is target selection, a topic still under debate due to the complex clinical profile presented by GTS patients (Porta et al. 2009b; Schrock et al. 2014). The most common target has been the thalamus, which arguably has also been reported to deliver the most efficacious overall outcomes in response to stimulation. In fewer cases, the globus pallidus internus has been targeted. Stimulation of this nucleus has been shown to be effective for tic reduction. In a small number of procedures, the anterior limb of the internal capsule and nucleus accumbens (ALIC/NA) have been targeted in order to address the behavioral aspects or comorbidities (in particular OCD) associated with GTS and/or as “rescue” surgeries (Servello et al. 2009). Owing to its generally successful outcomes and proposed reversible nature, DBS has become not only a preferred surgical technique, but also a promising option for the treatment of refractory GTS (Porta et al. 2009b; Maling et al. 2012; Schrock et al. 2014).

In addition to a direct therapeutic role, the advent of DBS provides a unique and powerful platform for investigation. Recordings from DBS targets can provide substantial insights into the neuropathophysiological mechanisms of GTS (Maling et al. 2012; Priori et al. 2013; Israelashvili et al. 2015). Acute single unit and local field potential (LFP) recordings from the thalamus reveal bursting behavior, consistent with reports from thalamic nuclei in various other neurological disorders (Priori et al. 2013). Chronic recordings from intra-thalamic networks demonstrate the effects of DBS on the dynamic changes in the spectral characteristics of LFPs in correlation with clinical benefit (Maling et al. 2012). For patients responding to therapy, a decrease in tic expression has been correlated to an increase in gamma oscillations (Maling et al. 2012). Additionally, changes in the cross-frequency coupling of theta-gamma band activity have been correlated with active improvement in symptomatology (unpublished results). These data provide evidence for the functional relevance of oscillations in the pathophysiology of GTS and support the role of the thalamus as a pivotal component in GTS dysfunction.

## Tic Expression

The cardinal feature of GTS is tics. As part of the fundamental dysfunction of the syndrome, they are nearly automatically described as chronic and involuntary; however, closer examination of when and how they occur adduces a far more intricate definition. Examination of their transient stability dynamics as well as the volition driving the act of ticcing will provide not only a better understanding, but a more definitive characterization of them within the confines of the disorder.

Tics fluctuate on both a short and long time scale across their dimensional features (intensity, frequency and severity). On short time scales, both motor and phonic tics (together and independently) exhibit short-term periodicity and “burst-like” behavior, often described as “bouts” (Peterson and Leckman 1998). Over longer epochs, these fluctuations are expressed as waxing and waning. Rather than being random, tic intervals exhibit structure in their dynamics, such as self-similarity; period doubling; and inverse power law scaling of the frequency distribution (with tic interval frequency plotted as a function of tic duration) (Peterson and Leckman 1998). The “bouts of bouts” experienced by GTS sufferers, therefore, are not the haphazard events commonly thought, but instead demonstrate the properties observed in non-linear dynamical systems. Within this framework, predictions of system behavior should be possible, where for example one could determine the dimensional features of tics across time scales. Clinically, this is nontrivial as the constant and seemingly erratic changes in tic expression (frequency, type, severity, complexity, number, location) can confound therapeutic strategies (Robertson 2000).

Overwhelmingly, ticcing is depicted as an action which is performed in response to the strong somatosensory sensations which generally precede a tic (Leckman et al. 1993; Woods et al. 2005; Leckman et al. 2010). As such, these premonitory urges are both clinically important and scientifically relevant for understanding GTS. The bodily sensations of such phenomena correspond with the anatomical region of the impending tic and are described as a “need”, “itch”, or “ache” (Woods et al. 2005; Marsh et al. 2007; Doyon et al. 2009; Leckman et al. 2010). Insofar as they are a disturbance heralding an impending dysfunctional state, they can be considered analogous to the aura experienced by epileptics prior to a seizure. However, premonitory urges are corporeal sensations rather than a perceptual disturbance; occur typically on fast time scales and in circumscribed areas; and elicit bodily responses to relieve them. Such impellent urges are representatively described by one subject as a feeling that she would “burst” unless she was able to “physically tic” (Leckman et al. 1993).

This brings into sharp focus the dynamics of tic expression and elucidates the ongoing debate about whether tics should be classified as voluntary, involuntary, or unvoluntary/nonvoluntary (Singer 2005; Ganos et al. 2015b). Although the symptomatology of GTS is conventionally characterized as the involuntary expression of tics, it is the premonitory urges—which occur in over 80 % of patients—that are unequivocally involuntary (Leckman et al. 1993; Fahn et al. 2011; Cohen et al. 2013). The confounding factor is that the large majority of sufferers (>90 %) report that they partly or wholly respond to these involuntary premonitory urges with voluntary tics (Leckman et al. 1993; Woods et al. 2005; Doyon et al. 2009). In fact, subjects report voluntary ticcing regardless of tic severity, although a higher incidence of premonitory urges has been correlated with an increase in both tic severity and tic complexity (Ganos et al. 2015b). Thus, the conundrum becomes clear: is a voluntary response to an involuntary sensation truly voluntary when the urge is irresistible?

Perhaps more light can be shed on the topic by employing objective electrophysiological measures as opposed to subjective reporting. The Bereitschaftspotential (BP) is an electrophysiological recording representing “free will” and the initiation of voluntary movement (Kornhuber and Deecke 1965; Libet 1985). It represents the cortical contribution to motor planning and precedes movement. Volition is immediately preceded by activation of the supplementary motor area (SMA) and premotor cortex ~1.2 to 0.5 s prior to movement, followed by activation in the primary motor cortex ~0.5 s to immediately pre-action (Shibasaki and Hallett 2006). EEG recordings from GTS patients reveal a complete lack of the first phase and often a lack of the second phase of the BP during spontaneous ticcing, but express the BP during imitation tics in all cases (Obeso et al. 1981; Hallett 2001; van der Salm et al. 2012). This might clarify entirely the question of voluntariness, but there is a confounding factor: in some cases, movement made in response to external triggers does not express a BP (Papa et al. 1991). It may therefore be possible that the absence of the BP is a result of tic response to the premonitory sensory urge, which acts as an internal trigger.

Ganos et al. show a correlation between increased interoceptive awareness and the intensity of bodily sensations referred to as premonitory urges in GTS (Ganos et al. 2015b). One interpretation of this is that the perception of a premonitory sensation would be oversensitivity to an internal somatosensory signal. Given the theory that the discernment of voluntary action depends on a perceptual discrimination in relation to a given threshold level, internal signals unrelated to volition could be misconstrued as voluntary movements if the internal somatosensory signal is above said threshold (Ganos et al. 2015a, b). Premonitory phenomena have been associated with maturation and an increased awareness of interoceptive sensation (Rajagopal and Cavanna 2013; Ganos et al. 2015b). Younger children tend to not be aware of premonitory sensations and also do not report ticcing voluntarily.

The insula is implicated in interoception and bodily sensations and, together with the somatosensory and medial frontal areas, has been consistently shown to be overactive in tic generation in GTS patients (Tinaz et al. 2015). It has been suggested by Tinaz et al. in a study demonstrating differences in the connectivity patterns of the right dorsal anterior insula in GTS versus controls that the insula could be involved in the increased awareness of premonitory sensations, leading to the urge to tic (Tinaz et al. 2015). Interestingly, although high interoceptive awareness has been associated with greater premonitory urges, subjects with GTS exhibit lower overall levels of interoceptive awareness as compared with healthy controls (Ganos et al. 2015b).

Given these subjective and objective reports, and the clear struggle nonetheless to check such debilitating sensory phenomena, the term unvoluntary seems the most authentic when describing volition in ticcing. The awareness and role of internal signals in the body and the accurate characterization of tics—which are identified as unwanted behaviors—have important implications for the biological basis of the disorder and are clinically valuable, in particular when targeting therapies.

There is a disparity in the incidence of premonitory urges with respect to tic classification (Jankovic and Stone 1991; Leckman et al. 1993; Woods et al. 2005). Although dystonic tics are most often associated with them, significant differences have been found between the correlation of simpler motor (dystonic) tics and premonitory urges (Leckman et al. 1993; Jankovic 1997). For example, eye-blinking and mouth movements have been reported to be less frequently preceded by premonitory urges (~30 %) as compared with shoulder jerks or head/neck movements (~60 %) (Leckman et al. 1993). This pattern also appears in phonic tics, albeit to a lesser extent, where sniffing has been reported at an approximate 30 % frequency of co-occurrence, while vocal tics were around 40 %. Correlations have also been shown between tic frequency, intensity, and complexity and premonitory urges in older youths but not younger ones (Woods et al. 2005). Premonitory sensations are overall reported by both genders nearly equally (except notably in association with throat clearing tics where female subjects reported nearly double the frequency of males); occur mostly in the palm, shoulder, and midline abdomen, and throat; and are typically (40 %) felt in muscle, but can occasionally also be found in joints and skin (Cohen and Leckman 1992)..

Tic expression can be influenced by contextual variables from either internal or external environmental cues which can affect both their frequency and severity (Marsh et al. 2007; Conelea and Woods 2008; Capriotti et al. 2014). Classically, it has been reported that tic-exacerbating variables include fatigue, social activities, or any environment which increases stress or anxiety (Conelea and Woods 2008). Tic-ameliorating factors include being in a quiet comfortable environment, sleep, and focused attention on a task (such as music or athletic performance, or math) (O’Connor et al. 2003; Conelea and Woods 2008). These contextual antecedents are reported from case study or descriptive design studies, which do not control for threats to both internal and external validity and cannot reliably address causal relationship.

In contrast, single-case experimental design investigations are inherently constructed to address causality. They indicate the factors that most influence tic expression: overt observation and the presence of others; reading and academic tasks; and tic related conversation and verbal instructions to suppress tics (Conelea and Woods 2008). In the latter case, verbal instructions resulted in reduced tic frequency in nearly half of the subjects with no apparent rebound effects (Meidinger et al. 2005). Interestingly, vocal—but not motor—tics increased in the tic-related talk condition (Woods et al. 2001). This is consistent with the suggestion that in some cases, tic location may be functionally related to the engaged activity (O’Connor et al. 2003). Similarly, tics were reported to occur more frequently during less challenging reading tasks or conditions (Watson et al. 2005), indicating focused attention is in fact relevant for amelioration of tics. Finally, in a large cohort of individuals with Chronic Tic Disorder, it was shown that overt observation (with a camera) significantly increased tic frequency when compared to covert observation (hidden camera) (Piacentini et al. 2006). Notably, the overt observation resulted in increased ticcing regardless of the physical presence of another. Furthermore, tic expression rates were independent of setting (home vs. clinic), and remained remarkably temporally stable across locations. The results of these studies provide empirically confirmed findings which are non-trivial for symptom management and evaluation in clinical practice.

## Genetic Factors

Tourette syndrome likely arises from complex genetic features that interact with environmental factors (Leckman et al. 1997). Transmission has been shown to be autosomal dominant, polygenic, and bilineal (Pauls and Leckman 1986; Hanna et al. 1999; Ali et al. 2013). One of the main risk factors is male gender, as it is 3–5 times more prevalent in boys as compared with girls (Kadesjo and Gillberg 2000). The disorder is highly heritable: (1) the incidence of GTS increases 10–100 fold if a primary relative has the disorder, (2) the incidence of GTS increases 5–20 fold if a primary relative has chronic tic disorder, and (3) studies of monozygotic and dizygotic concordance have reported between 50–94 % and 10–56 %, respectively, if one sibling had GTS and/or tic disorder (Price et al. 1985; Hyde et al. 1992).

Despite a strong hereditary component, Tourette syndrome presents with a range of clinical phenotypes and is therefore often considered to be a genetically heterogeneous disorder (Jankovic 2001; Grados and Mathews 2009; Ali et al. 2013; Cavanna and Rickards 2013). Presently, the most prominent chromosome aberration is associated with the SLITRK1 gene on chromosome 13 (Abelson et al. 2005). This gene has been shown to control neurite outgrowth and is expressed in the cortex, thalamus and basal ganglia, all areas intimately implicated in the pathophysiology of Tourette syndrome (Abelson et al. 2005; Proenca et al. 2011). Another locus of interest is on chromosome 15, the l-histidine decarboxylase (HDC) gene (Ercan-Sencicek et al. 2010; Karagiannidis et al. 2013; Castellan Baldan et al. 2014). The enzyme expressed by this gene converts l-histidine to histamine, which in turn regulates neurotransmission. Clearly, any alteration in this gene could lead to dysregulation of the information processing necessary for proper motor, cognitive, and executive function. Indeed, observations of HDC-deficient related symptoms in humans were later supported by animal experiments, in which similar sensorimotor and behavioral symptomatology was reproduced in mice (Castellan-Baldan et al. 2014). Although these are the most promising leads in the search for the genetic underpinnings of the disorder, both of these genetic anomalies are thus far considered to be rare functional variants found in only a small proportion of the disease population.

Looking for more common forms of genetic variation which distinguish a substantial portion of the Tourette’s population from the population at large, the first genome wide association study (GWAS) for GTS was published in 2012 (Scharf et al. 2013). Although some aberrant genes were found, this investigation could not report any common genetic variants that could be identified as risk factors among about 1500 subjects with Tourette syndrome as compared to over 5000 without it (Scharf et al. 2013). These results suggest either a rare single gene or, more plausibly, a multifactorial genetic effect might be contributing to the etiopathology. A GWAS combining GTS and OCD published 1 year later confirmed true functional variants associated with gene expression levels in the brain for each disorder (Yu et al. 2015). Interestingly, it proposed that these highly comorbid disorders likely do not share a genetic architecture, but rather have a complex genetic relationship.

There may be inherent difficulties to isolating specific risk genes which would unequivocally link to a seemingly heterogeneous disorder such as GTS. The alternative technique of gene network analysis, which reconstructs functional gene networks from the susceptible genes in disease-associated loci, may address this issue. A current study using transcriptome analysis of post-mortem striatal tissue has identified significant changes in the expression of gene clusters in the brain tissue of lifelong sufferers with severe GTS (Lennington et al. 2016). Ten modules of co-expressed genes revealed 309 down-regulated interneuron-related genes and 822 up-regulated immune-related genes. No significant correlation was found between the expression of the two classes of transcripts, suggesting that they may independently contribute to GTS pathophysiology. Thus, decreases in the signaling of various classes of interneurons in the striatum as well as aberrant activation of the immune system could each be contributing factors to dysfunction.

## Anatomy and Pathophysiology

In addition to genetic studies, electrophysiological, imaging and modeling investigations have provided many new insights into the possible pathomechanism(s) of GTS; still, its etiopathology has yet to be elucidated. Nonetheless, it is believed that Tourette syndrome results from dysfunction in the cortico-basal ganglia–thalamo–cortical network (CBGTC) (Leckman et al. 1997; Mink 2001). The circuits of the CBGTC are reported to control motor, emotional, and adaptive function as well as the ability to reason (Schultz 2004; Leisman et al. 2014). Although its precise mechanisms are still being actively investigated, impairment has been suggested to be architectural, as observed in altered connectivity patterns; physiological, as reported in abnormalities of dopamine transmission; morphological, as noted in the structural changes in the striatum and/or various regions of the cortex; metabolic, as revealed in cortical PET scans; or functional, as demonstrated by changes in the synchronization of neuronal ensemble activity (Wong et al. 2008; Draganski et al. 2010; Maling et al. 2012; Cui et al. 2014; Shprecher et al. 2014).

The CBGTC is purported to be essential for action gating and the conversion of goal-directed behavior to automated behaviors (Graybiel 2005; Doyon et al. 2009; Haber and Calzavara 2009; Draganski et al. 2010). The basal ganglia are involved in learning and memory and essential to optimal control of action (Graybiel 2005). With afferents from nearly all the functional subdivisions of the cortex as well as connections to motor; prefrontal; parietal; and temporal associative cortical areas via substantial efferents from its output nuclei to the thalamus, this largest subcortical structure in the human forebrain, which also spans the midbrain and diencephalon, is well-positioned to integrate motor, cognitive, and affective-motivational processes (Graybiel 2000; Hammond et al. 2007).

Dysfunction in the basal ganglia is clinically expressed in a range of movement and neuropsychiatric disorders, and in addictive states (Graybiel 2005). Anatomic and/or functional pathology in this network could understandably give rise to the signs and symptoms observed in GTS and GTS-related neurobehavioral disorders such as OCD and ADHD. Functionally, abnormalities in the associative and limbic circuits of the network have been associated with OCD, while structurally, reduced volume in the globus pallidus has been associated with ADHD (Aylward et al. 1996; Worbe et al. 2012). GTS patients exhibit both structural and functional alterations involving multiple re-entrant pathways of the CBGTC (Leckman et al. 2010).

The predominant models for dysfunction have been based on either insufficient inhibitory motor control due to decreased inhibitory output from the basal ganglia, which would result in frontal cortical hyperactivation as a result of thalamic disinhibition, or imbalance in the selection and suppression of competing motor programs in which desired behaviors are facilitated and unwanted ones are inhibited (Mink 2001; Albin and Mink 2006). Cortical dysfunction has also been proposed as a mechanism for GTS pathophysiology (Thomalla et al. 2014; Tinaz et al. 2014). In particular, the anterior cingulate cortex (ACC), insula and SMA have been implicated as they are believed to be important for urge and impulse control (Hallett 2015).

### Circuitry

**Fig. 1:**
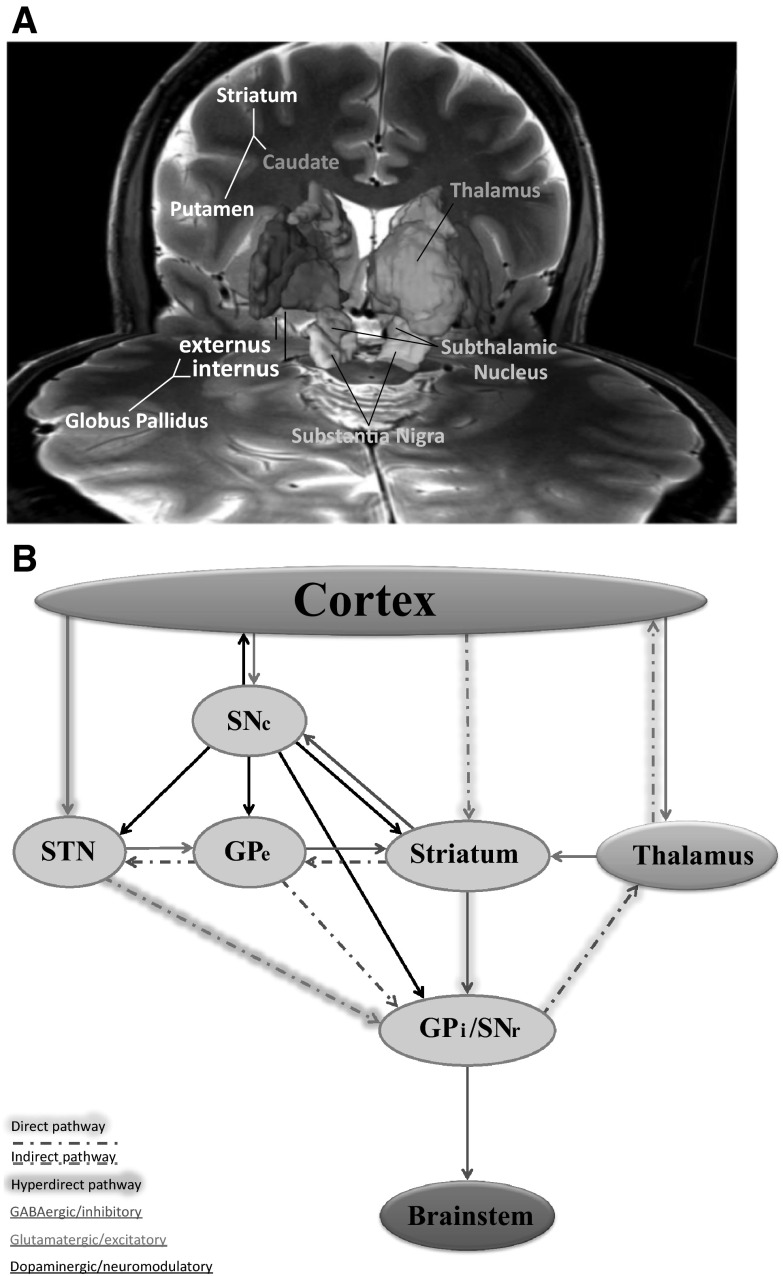
The cortico-basal ganglia–thalamo–cortical recurrent network. **a** Subcortical structures of the CBGTC network. Three dimensional visualization illustrates intimate topography of the subcortical constituents of the CBGTC. Figure modified from (Lenglet et al. 2012). **b** Schematic of the direct, indirect and hyperdirect pathways of the cortico-basal ganglia–thalamo–cortical network. A complex organization of highly interconnected structures orchestrates the cognitive, motor, and emotional processes underlying the repertoire of human behaviors. The striatum is the input nucleus for the basal ganglia (*beige circles*), which receives excitatory glutamatergic afferents from the cortex. It sends GABAergic inhibitory projections broadly to the both divisions of the globus pallidus and substantia nigra. The so-called “direct” and “indirect” pathways are based on the classification and connections of striatal efferents. The direct pathway (*highlighted in beige*) projection is monosynaptic to the GPi/SNr and expresses D1 dopamine receptors, substance P, and dynorphin. The indirect pathway (*dashed lines*) projects polysynaptically to the GPi/SNr and expresses D2 dopamine receptors, enkephalin, and neurotensin. The indirect pathway has a short and a long route. The short route affects the GPi/SNr via the GPe, while the long route adds a further connection with the STN before finally synapsing on the GPi/SNr. The hyperdirect pathway (*highlighted in purple*) bypasses the striatum, allowing the cortex to powerfully excite the GPi/SNr with fast conduction times. The activity of the entire basal ganglia is modulated by the dopaminergic cells of the SNc. *GPi* globus pallidus internus, *SNr* substantia nigra pars reticulata, *GPe* globus pallidus externus, *STN* subthalamic nucleus, *SNc* substantia nigra pars compacta. Copyright Rowshanak Hashemiyoon (Color figure online)

The central cortical and subcortical regions in the pathogenetic circuit include the (1) motor and associated motor, prefrontal, and limbic cortices; (2) striatum [caudate, putamen, and nucleus accumbens (NA)]; (3) globus pallidus [internus (GPi) and externus (GPe)], (4) substantia nigra [pars reticulata (SNr) and parts compacta (SNc)], (5) subthalamic nucleus (STN), and (6) thalamus (see Fig. 1a). The extrinsic connections of the pedunculopontine nuclei and the cerebellum may also be important for the neuropathological architecture of the disorder.

The cortex sends massive projections to the subcortex, with the striatum and STN as the receiving stations for the basal ganglia (see Fig. 1b). The striatum receives cortical afferents that are both convergent (from multiple anatomically separate but functionally related cortical areas) and divergent (one cortical area projecting to multiple striatal locations) (Flaherty and Graybiel 1991). It also receives substantial afferents from the intralaminar and ventrolateral thalamic nuclei and the SNc in addition to intrastriatal inputs. The striatum is thus a regulatory center in which information can be integrated, filtered and transformed. The GPi/SNr, which are considered to be a single structure divided by the internal capsule, are the basal ganglia output station. They further integrate inputs from other basal ganglia components and send their projections predominantly to the thalamus, but also to brainstem nuclei (Nambu 2015). The GPe connects these input and output stations, and the SNc modulates the network (Bar-Gad and Bergman 2001; Rivlin-Etzion et al. 2006).

**Fig. 2:**
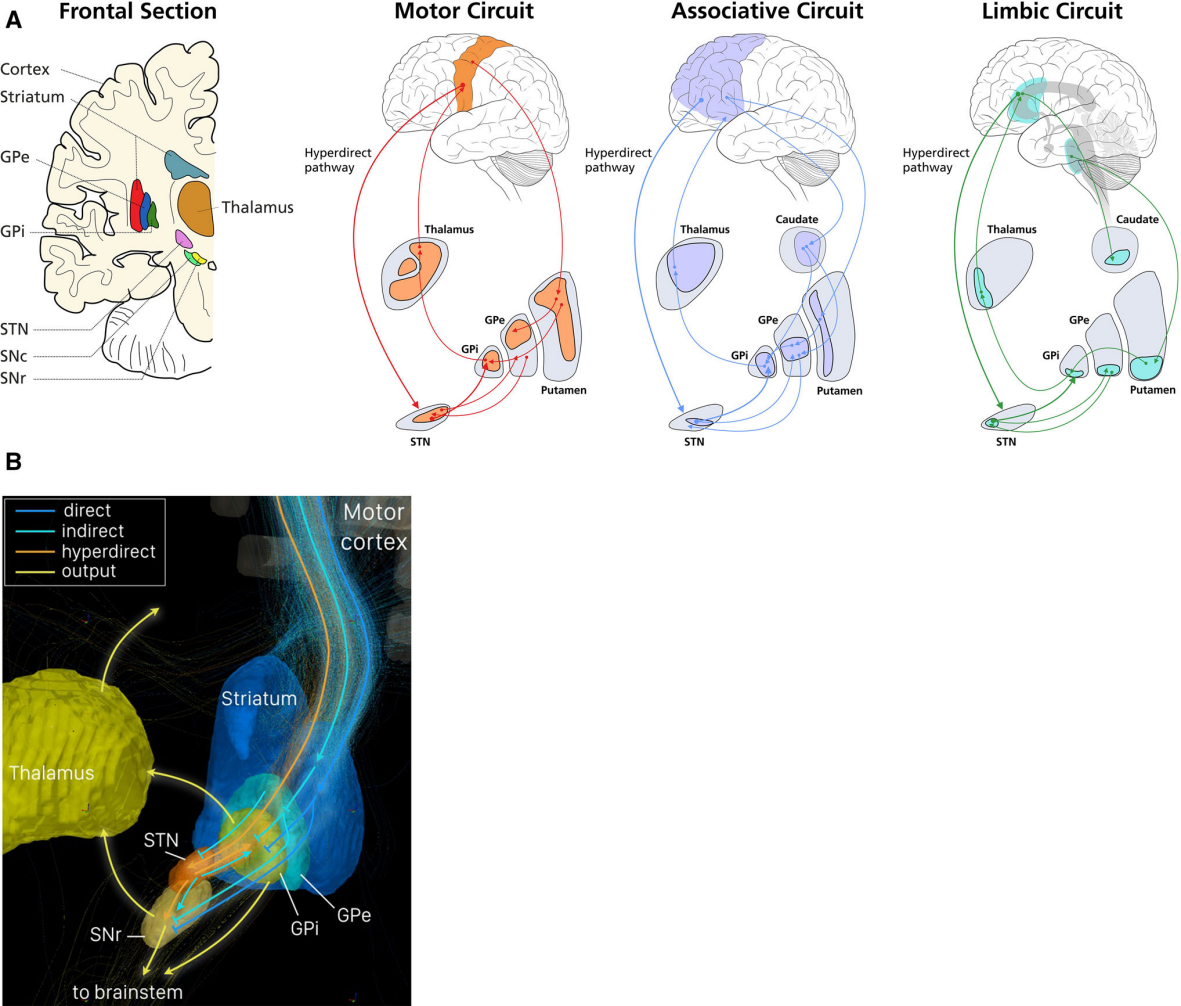
Anatamo-functional organization of the circuits of the CBGTC network. **a** Re-entrant circuits of the CBGTC form functionally and anatomically distributed, parallel circuits which integrate to perform massive processing in different brain areas collectively serving the same function. Disturbances are observed in motor, limbic, and associative functions in Tourette syndrome. The motor loop is derived from the primary motor, premotor, and supplementary motor cortex; the associative loop is derived from the dorsolateral prefrontal cortex; the limbic loop is derived from the anterior cingulate and orbitofrontal cortex. Figure modified from (Volkmann et al. 2010). Anatomical abbreviations as in Fig. 1b. **b** Anatomical representation of the human motor CBGTC with the direct, indirect and hyperdirect pathways illustrated using diffusion tensor imaging. All pathways originate from the cortex and make variable successive (and some branched) synaptic connections en route to the GPi/SNr. The thalamus receives organized, synthesized information from the GPi/SNr. In turn, it performs further processing and integration of impulses before conveying such to the cortex. *Arrows* indicate excitation while *vertical bars* indicate inhibition. Data collected from thirty subjects participating in the human connectome project; rendering generated with TrackVis software with subcortical structures based on the Harvard-Oxford and Atlasing of the Basal Ganglia atlases. Modified from Andreas Horn (own work) [CC BY-SA 4.0 (http://creativecommons.org/licenses/by-sa/4.0)

The CBGTC consists of multiple, parallel circuits named for their cortical areas of origin and action (see Fig. 2a) (Wichmann and DeLong 1996). The prototypical structure is one in which somatotopically specific projections from a designated frontal cortical area synapse onto their corresponding basal ganglia targets, which in turn project topographically to the thalamus; the circuit is completed by topographically maintained thalamocortical projections. Thus, each functionally distinct cortical area synapses onto its corresponding functional subdivision of subcortex and this organization is faithfully preserved throughout the circuit. The major circuits include the motor, prefrontal/associative, limbic, oculomotor, and orbitofrontal loops.

In general, motor circuits compose the dorsolateral division and limbic circuits travel through the ventromedial regions. As projections from the frontal cortex follow topographic organization through the subcortex, each circuit is devoted to its discrete behavioral function. These discrete circuits were initially thought to be structurally and functionally segregated and thus “closed”; however, some intermixture between these pathways has been subsequently suggested, and is necessary for the performance of such tasks as the learning and execution of appropriate behavioral responses (Alexander et al. 1986; Joel and Weiner 1994; Haber and Calzavara 2009). Integration within the network is the foundation upon which motivation is translated into movement-requiring, goal-oriented behavior and, ultimately, habit formation (Frank 2009; Maia and Frank 2011; Moustafa et al. 2014).

The CBGTC consists of mono- and poly-synaptic “direct” and “indirect” pathways (see Fig. 2b) which are organized topographically and distinguishable histologically (Kalanithi et al. 2005; Haber and Calzavara 2009). Historically, the direct and indirect pathways were believed to effect the mechanism of motor control by tandem operation. In the direct pathway, excitatory input from the cortex activates inhibitory output from the striatum which suppresses the spontaneously active basal ganglia output nuclei’s (GPi/SNr) inhibitory output, thus disinhibiting or releasing the thalamus (see Table 1). This is the mechanism suggested for motor program selection. By modulating inhibition at multiple levels, the indirect pathway delivers a net excitatory effect on the GPi/SNr, causing thalamic inhibition and the suppression of alternate, unwanted motor behaviors.

**Table 1:** Pathways and their neurotransmitter effects

Pathway	Major synaptic neurotransmitters	Effect on GPi/SNr	GPi/SNr output	Thalamic output
Direct	Glutamate, GABA	Inhibitory	↓	↑
Short indirect	Glutamate, GABA, GABA	Disinhibitory	↑	↓
Long indirect	Glutamate, GABA, GABA, Glutamate	Excitatory	↑	↓
Hyperdirect	Glutamate, Glutamate	Excitatory	↑	↓

Major neurotransmitters released in the pathways of the CBGTC at their synaptic target nuclei. All pathway effects are presented beginning with the cortico-striatal synaptic connection and ending in the GPi/SNr. Activity in the GPi/SNr inversely affects thalamic activity

This dualistic model eventually gave way to a new tripartite theory which includes the “hyperdirect” pathway. In the hyperdirect pathway, information is monosynaptically communicated from motor and non-motor cortical areas to the STN, bypassing the striatum. Information can then travel to the thalamus with faster conduction times via direct efferents from the STN to the GPi/SNr. This scheme creates a triphasic temporal effect on the thalamus (Nambu et al. 2002). Via the fast conducting hyperdirect pathway, the thalamus is initially inhibited; it is then subsequently disinhibited by the direct pathway, with the indirect pathway finally exerting inhibition once more. This arrangement could allow for precise selection of action, with a circumscribed initiation and termination.

**Fig. 3:**
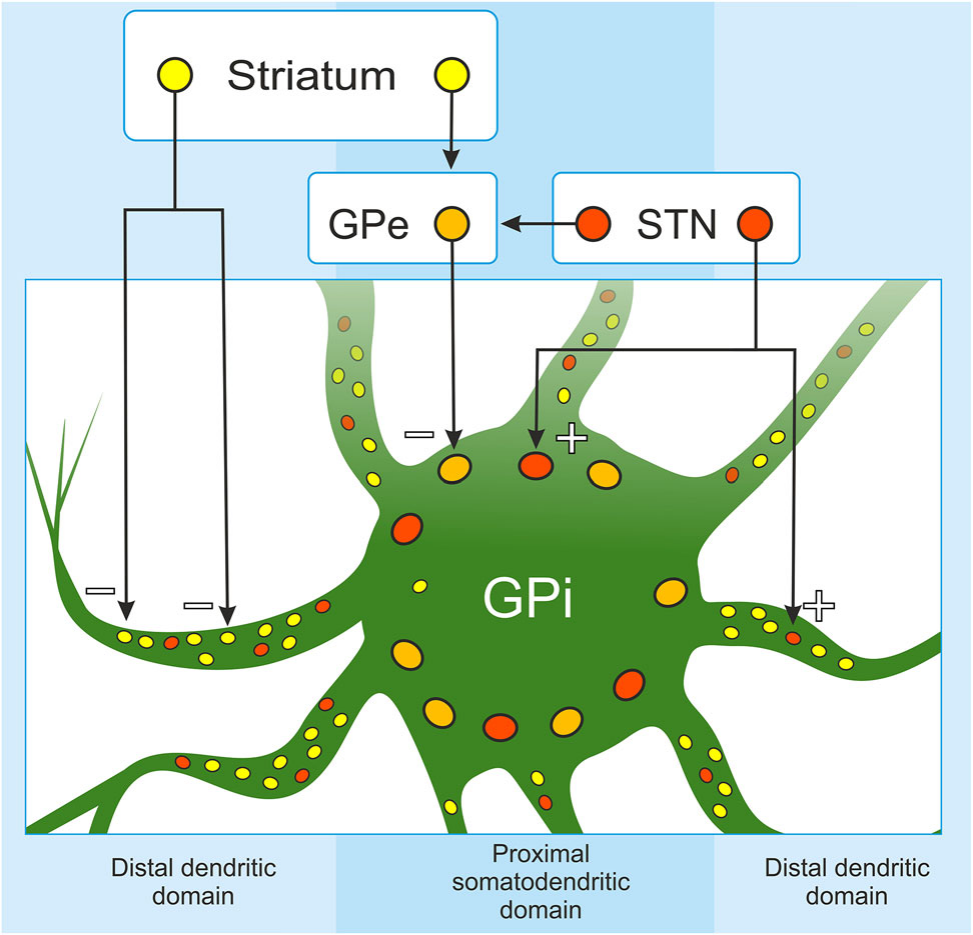
Characterization of basal ganglia synapses. The GPi receives input from basal ganglia nuclei via the direct and indirect pathways. It has been shown that both the strongly inhibitory cells of the striatum and the powerfully excitatory cells of the STN send efferents converging upon single pallidal cells (Hazrati and Parent 1992a). The micro-architectural arrangement of these inputs is an important determinant for the integration and transformation of information. Direct input from striatal efferents closely embraces distal GPi dendrites (*yellow circles*) while their polysynaptic input via the GPe forms a tight pericellular arrangement on the somato-dendritic domain (*orange circles*). The latter produce a strong inhibitory effect because of their size and location on the post-synaptic pallidal neurons. STN-pallidal (both GPi and GPe) fibers produce a strong excitatory effect directly on the cell bodies (*red circles*), but also exert some influence on pallidal dendrites with their collaterals that loosely entwine them. The small terminals expressed by the STN on distal GPi dendrites are much fewer than the striato-pallidal synapses by comparison. Figure modified from Hazrati and Parent (1992a) (Color figure online)

**Fig. 4:**
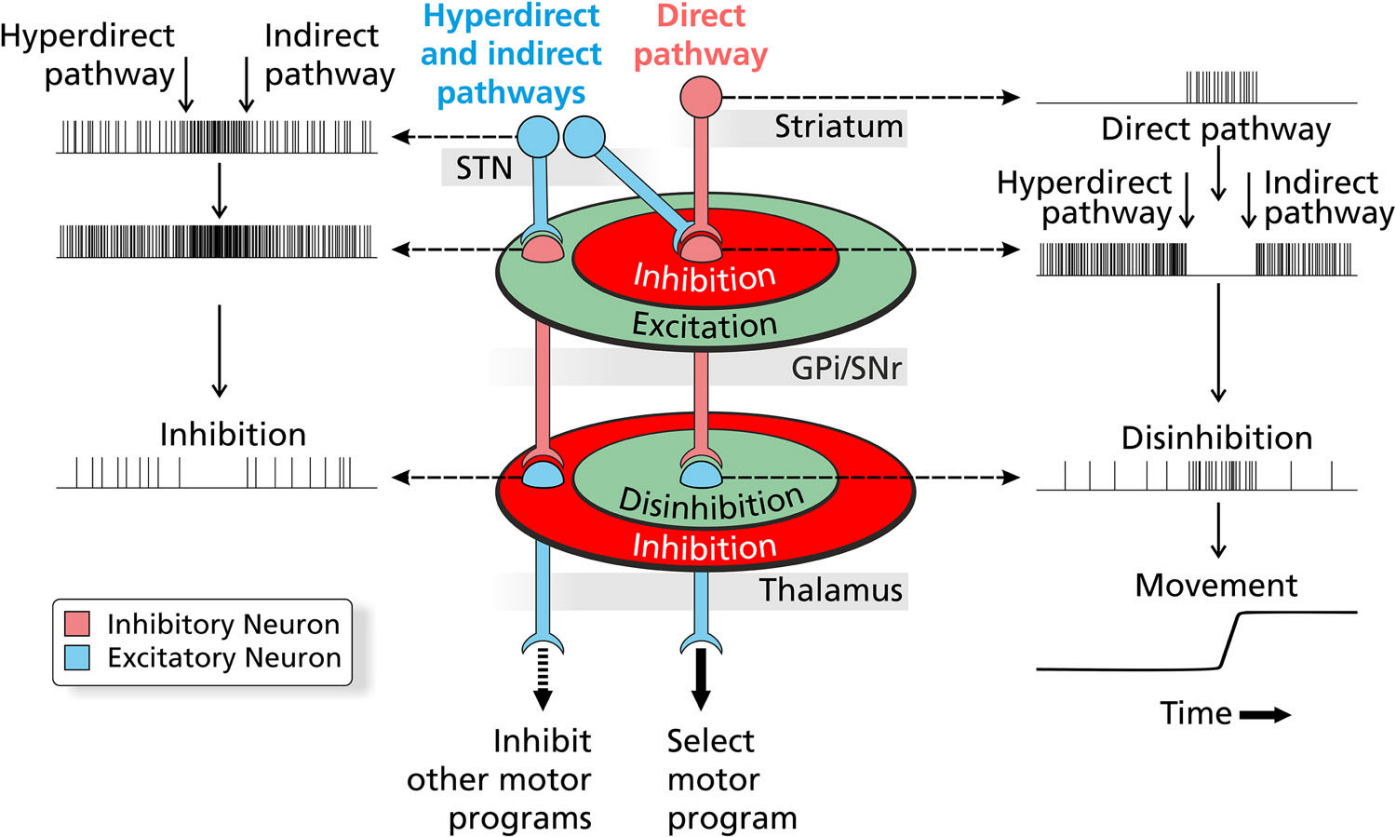
Center-surround model for action selection. Schematic diagram represents the contribution of the direct, indirect and hyperdirect pathways to motor action selection. Cortical input travels serially from the striatum to the GPi/SNr to the thalamus in the direct pathway (*center figure*). Inhibitory GABAergic input from the striatum to the spontaneously active pallido-nigral target results in disinhibition of the thalamus, allowing it to activate the cortex and select the desired motor program. Sequential effects of cortical input on the spiking activity of these structures in the direct pathway are represented in the *right panel*. Excitatory STN input from the hyperdirect and indirect pathways—working on both a faster and slower time scale, respectively—has broad spatial effects on the GPi/SNr. These pathways are suggested to effectuate precision initiation and termination of selected motor programs in addition to inhibiting unwanted/competing ones. Effects of cortical input on spiking activity in the hyperdirect and indirect pathways are represented in the hypothetical time series in the *left panel*. The hyperdirect pathway is represented to the *left* of the indirect pathway in *both panels* to indicate timing effects on the neuronal stream. The physiology and microanatomy of the circuit provide a temporal and spatial organization which tightly controls action gating. Figure modified from Nambu (2008)

In addition to this temporal organization, communication between nuclei of the CBGTC also exhibits a distinct spatial organization due to differential anatomical connections. An example of this is the powerful striatal and STN efferents which are of opposite polarity, but converge upon individual pallidal neurons (in both segments of the globus pallidus). Although converging on a single cell, the STN-pallidum fibers provide broad arborizations synapsing onto the proximal dendrites of pallidal neurons with highly branched collaterals closely surrounding their somata, while the distal end of striatal-pallidal fibers closely embrace pallidal dendrites (see Fig. 3)  (Hazrati and Parent 1992a, b). This contributes to a “center-surround” organization (see Fig. 4) (Nambu 2007).

The configuration of this dynamic spatio-temporal model is purported to actualize the *coordination* of voluntary actions (Nambu et al. 2002; Haber 2008). Desired actions are allowed by focused activation (via disinhibition) on a longer timescale of the circumscribed center area of thalamic neurons via the direct pathway. In contrast and in parallel, the hyperdirect and indirect pathways, involving subthalamo–pallidal connections, produce extensive activation of the output nuclei of the basal ganglia, resulting in inhibition of large areas of thalamus and the cancellation of unwarranted, competing motor programs. The physiology and microanatomy of the circuit provide a temporal and spatial organization which tightly controls action gating. Activity imbalance in these pathways is shown to produce a range of hypo- and hyper-kinetic disorders, such as Parkinson’s and hemiballismus.

This imbalance between facilitation and inhibition has also been implicated in habit formation. The conversion of a desired motor program (goal-directed behavior) into a habit (automated responses) requires proactive inhibitory control and the participation of the associative and motor circuits of the CBGTC, respectively (Aron 2011; Jahanshahi et al. 2015). The suppression of tics in response to premonitory urges postulates the imposition of goal-directed inhibition. Investigations of spontaneous versus imitation tics suggest the former are due to either a reduction in the activation of the mechanisms of habitual inhibition or hyper-active generation of habitual actions (Jahanshahi et al. 2015).

**Fig. 5:**
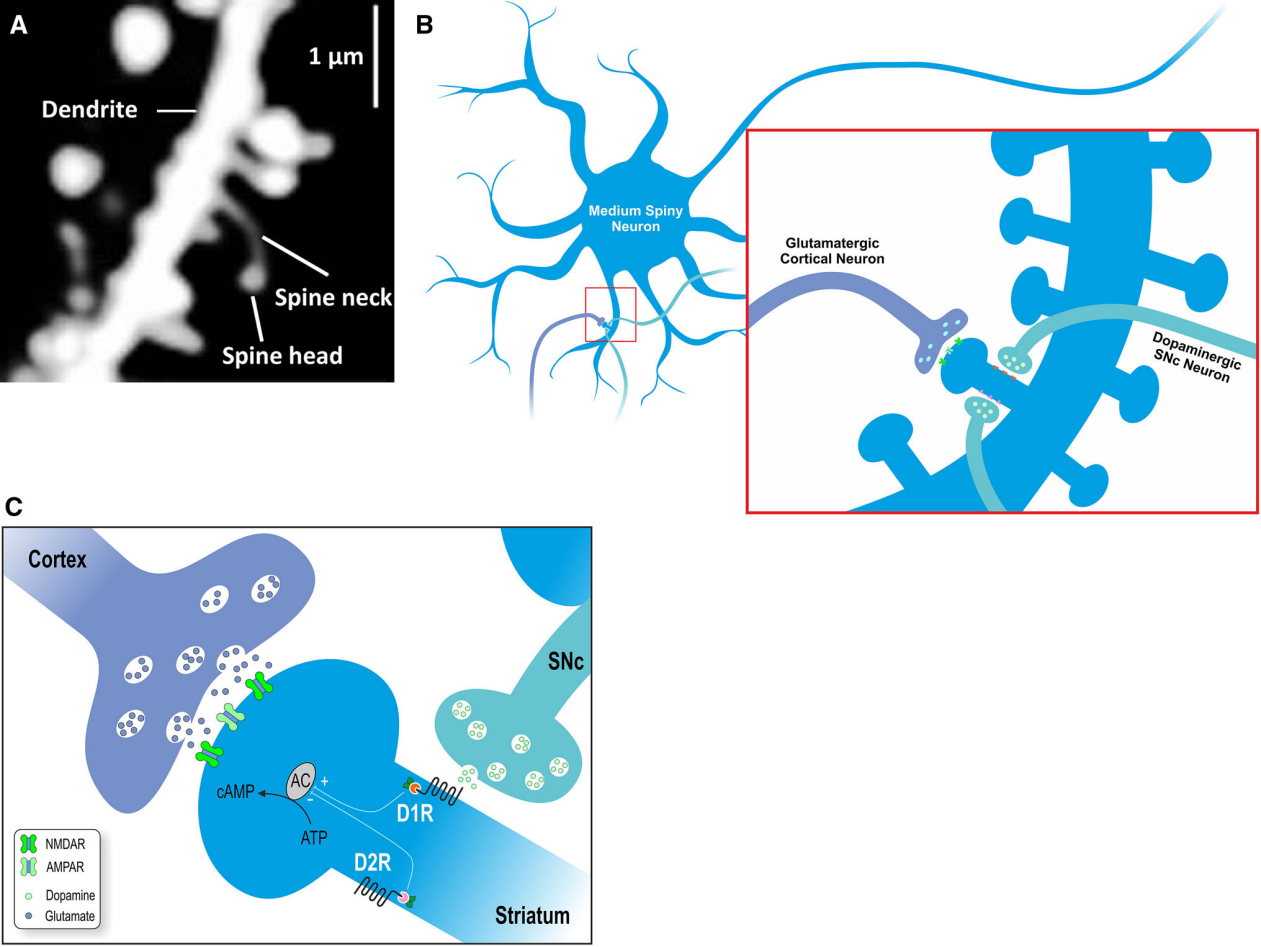
Neuromodulation of striatal afferents and efferents. **a** Medium spiny neuron. Image of medium spiny neuron (MSN) showing dendritic spines, with spine neck and spine head clearly identified. Neuron with Enhanced Green Fluorescent Protein imaged using laser scanning two photon microscope. **b** Neuromodulation of excitatory glutamatergic cortico-striatal input by dopaminergic nigro-striatal input. Cortical neurons make excitatory glutamatergic synapses on the heads of the dendritic spines of striatal MSNs. The dopaminergic SNc neurons exert modulatory control over cortico-striatal connections by synapsing on the necks of MSN spines, affecting the efficacy of cortical inputs via the widespread distribution of dopaminergic receptors on the cell membrane. Additionally, the striatum is the primary target of dopaminergic signals; thus, it is well-positioned to regulate cortico-striatal plasticity. **c** Schematic diagram showing micro-anatomical arrangement and associated biochemical interactions of cortical, SNc and striatal neurons. This organization is the mechanism by which dopamine exerts modulatory control over the activity of the direct and indirect pathways and thus regulates behavior. Striatal MSNs of the direct pathway are differentially excited by D1 receptors which activate adenylyl cyclase, while MSNs of the indirect pathway express predominantly D2 receptors which inhibit adenylyl cyclase. Tic expression may be the result of excessive activation of the direct versus indirect pathway, explaining the success of D2 antagonists in relieving tics. The interactions of these neurons at the level of the synapse can result in plasticity, such as occurs as a result of activation of AMPA/NMDA and DA receptors. *D1R* D_1_ subtype of the dopamine receptor, *D2R* D_2_ subtype of the dopamine receptor, *NMDAR* N-methyl-D-aspartate receptor, *AMPAR* α-amino-3-hydroxy-5-methyl-4-isoxazolepropionic acid receptor, *AC* adenylyl cyclase, *ATP* adenosine triphosphate, *cAMP* 3′,5′-cyclic AMP

The modulatory role of dopamine (DA) in the CBGTC is crucial for learning habits and for driving the transition between goal-directed behavior and habit formation (Wong et al. 2006). Reinforcement learning is suggested to occur in part due to the dopaminergic cells of the SNc. These neurons code reinforcement prediction errors which signal the difference between observed versus predicted events (Nambu 2008; Maia 2009). Such salient stimuli direct the responsiveness of striatal projection neurons, in part due to the ability also of the SNc to alter synaptic efficacy at the cortico-striatal synapse (see Fig. 5) (Leckman et al. 2006; Nambu 2008). This reinforcement of salient stimuli contributes to habit formation.

**Fig. 6:**
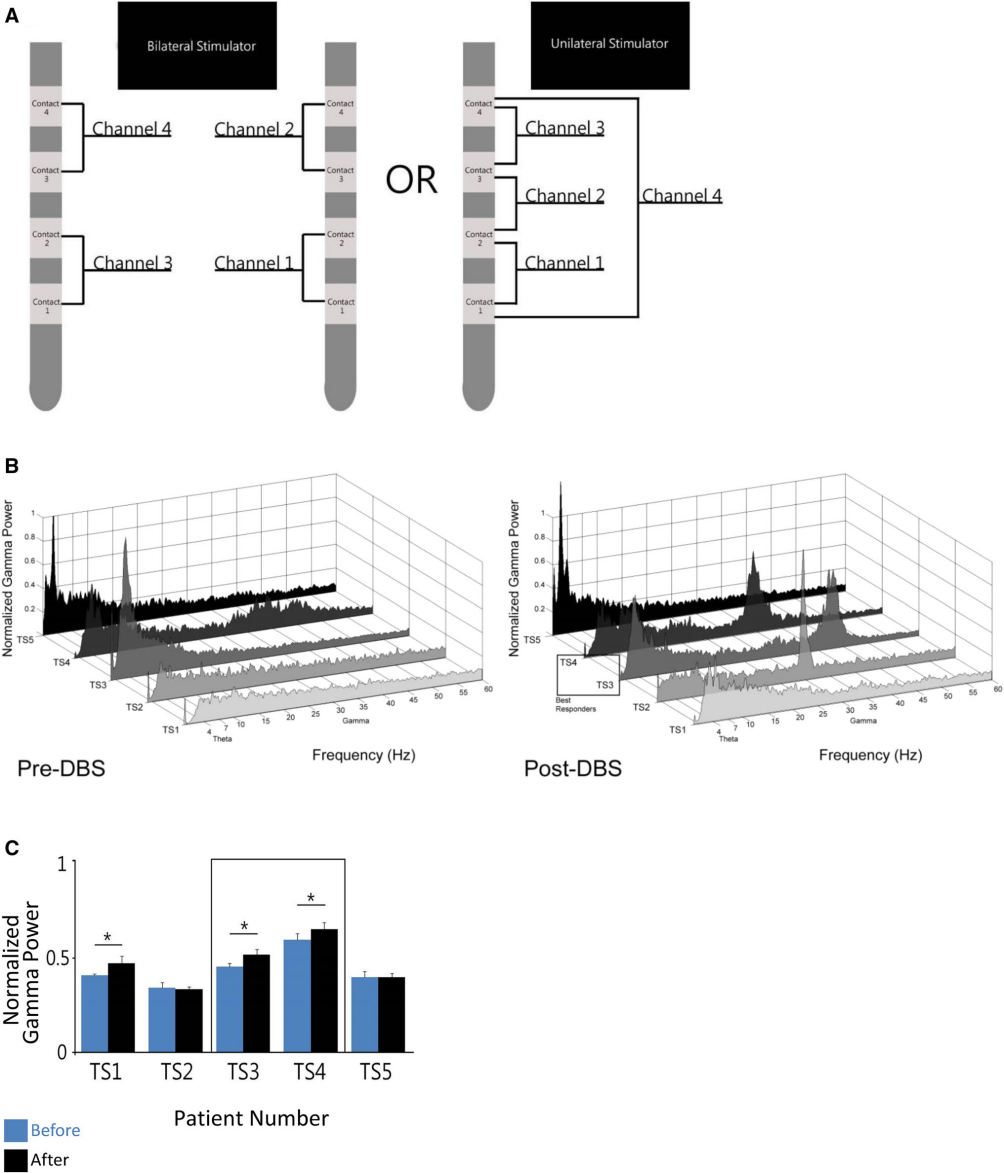
Increased gamma power correlates with symptomatology. Comparative power spectra before and after chronic DBS therapy for GTS are shown. Bipolar local field potential recordings are taken from the DBS macroelectrode, chronically implanted in the CM thalami of five patients. In order to faithfully represent the thalamic network, the largest sampling of the neuronal population is taken (channel 4 in all subjects; TS2 has a bilateral stimulator). A correlation was found between increased synchronization of neuronal ensemble activity in the gamma band and improved symptomatology. Figure modified from (Maling et al. 2012). **a** DBS macroelectrodes. Channel configurations for electrode recordings are shown. Channel four has the largest sampling area, representing a larger network of neuronal activity. **b** Electrophysiological recordings from five patients 1 month post-operatively (stimulator not yet turned on) (*left graph*) and after 5 months (*right graph*). Motor and vocal tics were assessed using the YGTSS. Patients who responded with significant symptomatological improvements, reflected in lower YGTSS scores, (responders), also showed significant increases in the spectral power of local field potentials in the gamma range, indicating that GTS is a disorder of *hyposynchronization* and that gamma oscillations may be important to the pathophysiology of GTS. After several months, patients one and five did not show significant clinical benefit (or change in YGTSS) and displayed aberrant gamma synchronization (as evidenced by the continued absence of gamma peaks over time). Variation was found in the final lead (electrode) location as compared with the others. The absence of a normal oscillatory regime combined with electrode location disparities suggests that the stimulation of the CBGTC network at the level of the CM thalamus addresses the pathophysiological basis of GTS and “corrects” the resulting dysfunction in signal transmission. **c** Acute stimulation correlates with increase in gamma synchronization. Immediate increase in thalamic gamma power was observed in response to acute DBS stimulation in long term responders, indicating a physiological importance for the role of gamma oscillations. Because changes in synchrony are immediately observed in those who also derive the greatest benefits from therapy chronically, gamma oscillations may be critical to the pathophysiology underlying GTS and might be used as a biomarker. Stimulus–response changes in gamma may be useful intra-operatively in determining optimal target localization

Children and adults with GTS present with impaired habit learning such that rate of learning inversely correlates with tic severity (Marsh et al. 2004). Perturbation of DA neurotransmission and decrease in intrastriatal inhibition are factors that could give rise to each, respectively. Such pathologies have been reported. The efficacy of DA antagonists in relieving tics has led to a predominant theory of DA dysfunction, and various studies report structural changes in the striatum, including a decrease in the number of parvalbumin-immunoreactive striatal interneurons (Kalanithi et al. 2005; Kataoka et al. 2010; Buse et al. 2013).

There is much support for CBGTC in the pathophysiology of Tourette, but other brain regions may also engender GTS dysfunction. Numerous other areas have been targeted as regions of interest in GTS, either via direct interaction with the CBGTC or through different mechanisms. Foremost among these is the cerebellum, owing to its role in motor function as well as learning and plasticity (Tobe et al. 2010). Unlike basal ganglia diseases which are associated with impairment of involuntary movement, cerebellar disorders are associated with failing of voluntary movement. The role of the cerebellum in the pathophysiology of GTS was most recently supported in an investigation in which vocal and motor tics were induced by focal disruption in ventral and motor striatum, respectively (McCairn et al. 2016). Interestingly, metabolic observations in those experiments also implicated the hippocampus and amygdala, although the other subcortical nuclei of the CBGTC were not associated. Additional implicated nuclei include the pedunculopontine, with its reciprocal connections to the STN, and dorsal raphe, due to their projections to DA neurons.

### Pathology: Anatomic

Prevailing theories suggest the disturbances observed in GTS and its associated comorbidities arise as a consequence of disturbances in the structural and functional components of the CBGTC-namely the cortex, basal ganglia and thalamus. Alteration of brain morphological parameters in adults includes changes in both gray and white matter (Liu et al. 2013). Structural changes have been reported in the prefrontal cortex, limbic structures and basal ganglia of individuals with GTS, consistent with the dysfunction and natural history characteristic of the disorder (Singer et al. 1993; Hyde et al. 1995; Peterson et al. 2003; Bloch et al. 2005; Plessen et al. 2009). Reduction in caudate volume has been correlated with symptomatology in both children and adults (Hyde et al. 1995; Peterson et al. 2003; Bloch et al. 2005). Childhood caudate volume was observed to be a predictor of symptom severity in early adulthood (Bloch et al. 2005). In a longitudinal magnetic resonance imaging (MRI) study, Bloch et al. showed a strong inverse correlation between caudate volumes in children and tic severity and OCD symptomatology later in life. In contrast to this, an MRI study of monozygotic twins, concordant for the presence of GTS but discordant for its severity, while finding a slightly reduced right caudate volume in the more severely affected twin, nonetheless emphasized the proportional loss of ventricular asymmetry to the degree of clinical severity (Hyde et al. 1995). MRI in a large cohort of GTS sufferers demonstrated reduced bilateral cerebellar surface volumes proportionally correlated with increased tic severity (Tobe et al. 2010). For OCD comorbid cases, the results were reversed with increasing hypertrophy in the lateral cerebellar hemispheres bilaterally, correlated with progressively more severe OCD symptoms.

Various imaging studies have shown decreases in the volumes of the caudate, putamen, and globus pallidus. Neuropathological studies have also shown structural alterations. The sensorimotor caudate and putamen showed a 50–60 % decrease in the number of interneurons. In the pallidum, the GPi has shown increases in the total number of parvalbumin expressing neurons, while the GPe and caudate showed a decrease in the number and density of these cells (Kalanithi et al. 2005; Kataoka et al. 2010).

Careful examination of the literature reveals some inconsistencies and conflicting results using imaging and other indirect techniques. Such contradictions need to be examined for matching parameters or confounding elements. For example, conflicting reports of volume increase in the dorsolateral putamen could be attributed to other qualifying factors, such as comorbid effects (from ADHD, for example) or medication (Peterson et al. 2003; Draganski et al. 2010).

GTS subjects taking neuroleptics had significantly differing basal ganglia volumes when compared to healthy subjects or to those not on medication (Peterson et al. 2003). Subjects taking typical neuroleptics tended to have significantly larger caudate and globus pallidus volumes, with the putamen and ventral striatum showing the same trend (Peterson et al. 2003; Draganski et al. 2010). In contrast, subjects taking atypical neuroleptics, had slightly smaller caudate and putamen volumes, but larger globus pallidus volumes. This volume-modifying effect was not seen with α-adrenergic agonists, tricyclic drugs, or specific serotonin reuptake inhibitors.

These results should be appraised in the context of the known effect of neuroleptics on the basal ganglia. Independent of GTS, they are recognized modifiers of basal ganglia structure. For example, first generation (classic) antipsychotics cause increases in caudate volume, while second generation antipsychotics have the opposite effect. In fact, switching from the former to the latter can reverse the effects of the first generation antipsychotics, bringing volumes back to normal. Such drug-related effects on anatomical volumes should be considered when reporting and interpreting results, both to temper over-interpretation and to examine for drug-modified interactions with existing changes in brain volumes.

As a broad range of motor and limbic cortical regions are implicated in the dysfunctional network associated with GTS, examination of their morphological changes could provide insight into their role in the pathogenesis of GTS (Peterson et al. 2001; Sowell et al. 2008; Thomalla et al. 2009). Increased volume has been shown in dorsal lateral prefrontal and parieto-occipital regions with decreases in inferior occipital volumes (Peterson et al. 2001). Additionally, decreased grey matter volumes were also observed in the medial orbitofrontal, anterior cingulate and ventrolateral prefrontal cortices and operculum bilaterally (Draganski et al. 2010). Corpus callosum size was found to be inversely correlated with dorsal lateral prefrontal and orbitofrontal cortical volumes to a greater magnitude than found in controls, and positively correlated with tic severity (Plessen et al. 2004). In these studies, the size of the corpus callosum was shown to be smaller overall in children and larger in adults, correlating with the natural history of the disorder. Reduced callosal size would limit interhemispheric communication and result in diminished input to inhibitory neurons in the prefrontal cortices.

Draganski et al. reported a negative correlation between prefrontal cortical thickness reduction and tic severity (Draganski et al. 2010). In adults, primary somatosensory cortex and right dorsal premotor cortex showed increased cortical thickness. Increased cortical thickness and grey matter volume in the left primary somatosensory cortex and prefrontal cortex were also positively correlated with the intensity of premonitory urges. Morphological analysis of the sensorimotor cortices controlling movement and vocalization in affected children showed cortical thinning in the frontal and parietal lobes positively correlated with tic severity (Sowell et al. 2008).

Comorbidities and tic severity modulated grey matter volumes (Draganski et al. 2010). In particular, ADHD comorbidity conferred significant effects. ADHD comorbid subjects were observed to have substantial decreases in the left inferior parietal cortex and posterior hippocampus bilaterally, while OCD comorbidity was not correlated with any changes. ADHD comorbidity also determined the polarity of correlation between orbitofrontal cortex/ventrolateral prefrontal cortex thickness and tic severity, as assessed by the Yale Global Tic Severity Scale (YGTSS). Those with ADHD + GTS or ADHD + OCD + GTS were reported to show a negative correlation between orbitofrontal cortex/ventrolateral prefrontal cortex thickness and tic severity, while those with only OCD + GTS showed a positive correlation between thickness and tic severity (Draganski et al. 2010). Nucleus accumbens volume was negatively correlated with severity of OCD.

White matter analysis has shown widespread abnormalities in both children and adults with GTS. Tic severity has been reported to be inversely proportional with white matter volume in the right frontal pole and positively correlated with increases in axial diffusivity in children (Liu et al. 2013). These subjects also showed a positive correlation with mean diffusivity and tic duration. In adults, intensity of premonitory sensations was negatively correlated with fractional anisotropy (FA) values in the parietal portion of the superior longitudinal fascicle (Draganski et al. 2010; Liu et al. 2013). Adults also showed significant FA decrease in the genu of the corpus callosum, and FA increase with mean diffusivity decrease was observed bilaterally in the somatosensory area (Draganski et al. 2010). Significant axial diffusivity and mean diffusivity increases were reported in children in the anterior thalamic radiation, right cingulum bundle projecting to the cingulate gyrus, and forceps minor (Liu et al. 2013).

In a study using probabilistic tractography, as well as FA and radial diffusivity to examine the hypothesis that aberrant brain development affects the structural connectivity of the CBGTC in adults with GTS, Worbe/Marrakchi-Kacem et al. reported abnormally enhanced structural connectivity between the striatum and thalamus with primary motor and sensory cortices (Worbe et al. 2015). The striatum and thalamus also exhibited abnormal connectivity with the paracentral lobule, SMA and parietal cortices. In the subcortical aspect of the CBGTC, enhanced motor connectivity in the thalamo-putaminal tract showed strong, positive correlation with tic severity. Microstructural abnormalities of white matter were found in the pathways between the cerebral cortex, basal ganglia, and thalamus. Interestingly, the microstructural axonal abnormalities of the cortico-striatal pathways showed gender dimorphism as they were more prominent in females.

### Pathology: Metabolic

Metabolically, fMRI studies have reported increased activity in the paralimbic areas, SMA and parietal operculum prior to ticcing (Bohlhalter et al. 2006; Hampson et al. 2009). Of these, only the SMA was activated and showed a broad cross-correlation with the motor cortex in a natural versus imitation ticcing paradigm, suggesting a neurophysiological correlate for the premonitory sensations that precede and drive tic behavior (Hampson et al. 2009). This theory is supported by electrical stimulation of the SMA with subdural electrodes in human epileptic subjects, which generated both movement and the urge to move (Fried et al. 1991).

Bilateral SMA and lateral premotor cortex, as well as midline activity, were shown to increase in ^18^F-fluorodeoxyglucose PET measures, which also showed decreased metabolic activity in the limbic caudate and thalamus (Swain et al. 2007). In a similar study, no changes in glucose metabolism overall within and between the cerebral cortices were observed; however, the frontal and temporal lobes bilaterally showed a relative hypermetabolism in GTS patients (Chase et al. 1984). In particular, an inverse correlation was shown between cortical metabolism and severity of vocal tics in the middle and inferior portions of the frontal lobes bilaterally, while coprolalia was associated with the left parasylvian region. Additionally, the basal ganglia—predominantly the striatum—exhibited a 16 % greater glucose metabolism in GTS patients than controls.

An event-related [^15^O] H_2_O PET synchronized with audio- and video-taping exploring the duration and frequency of tics showed abnormal brain activation in a distributed neural system including regions in the neocortical, paralimbic and subcortical areas (Stern et al. 2000). Areas associated with sensorimotor, language, executive, and limbic functions such as the primary motor, supplementary motor, premotor, anterior cingulate, and dorsolateral-rostral prefrontal cortices; Broca’s area; insula; claustrum; putamen and caudate were highly temporally correlated with motor and phonic tics. These results support the theory that GTS pathophysiology consists of a large scale network that either spontaneously initiates motor and vocal behavioral repertoires, or fails to suppress them.

An investigation which also employed [^15^O] H_2_O PET in which vocal and motor tics were induced in non-human primates reported a much smaller network with different activated brain regions (McCairn et al. 2016). Injection of the GABA antagonist bicuculline into the NA or dorsolateral sensorimotor putamen produced repetitive complex vocalizations or motor tics in the orofacial and/or the arm region, respectively. In the limbic circuit, unilateral disinhibition of the NA significantly increased regional cerebral blood flow bilaterally in the anterior cingulate gyrus, the amygdala and hippocampus. In the motor circuit, this increase in blood flow was observed ipsilaterally (to injection) in M1 and contralaterally in the cerebellum post-putamenal injection. Motor tics were not concomitant with vocal tics and each circuit maintained its unique regions of increased activation for corresponding tic type. These results suggest: 1) vocal and motor tics are a resultant property of the interruption of GABAergic communication in specific striatal regions which produce circumscribed circuit-wide increases in activation and 2) focal interruption in the limbic/motor circuit does not necessarily lead to widespread shared dysfunction between the circuits in the larger CBGTC.

### Pathology: Functional

Significant alterations in the temporal patterning of neuronal activity in the pathological circuit reflect changes in information processing and may provide insight into the underlying neurophysiological mechanisms of dysfunction in GTS. The use of fractional amplitude of low frequency BOLD fluctuations in resting-state fMRI allows a quantitative measure with more focus on neuronal fluctuations and can be applied in the study of neurophysiology of neuropsychiatric disorders (Zou et al. 2008; Lai and Wu 2015). In such an investigation of drug-naïve, pure GTS (no comorbidity) children, significant decrease in functional activity was measured in the amplitude of low frequency fluctuations (ALFF) (0.01–0.1 Hz) and the fractional ALFF (fALFF) in multiple relevant cortical regions (Cui et al. 2014). Significant increase in the fALFF was observed subcortically, including in the left putamen and bilateral thalami. These results are consistent with the prevailing model of GTS dysfunction of excess striatal activity leading to increased thalamic activity and an overexcited neocortex. Further support for this was found in the observation that tic severity was positively correlated with increased fALFF in the thalamus, “confirming its putative role in the pathogenesis of TS.”

In humans, the centre médian/parafascicular nucleus of the thalamus (CM/Pf) is the primary source of thalamo-striatal projections, sending massive efferents to the dorsal striatum. The primate CM/Pf, via innervation of the nucleus accumbens; caudate; putamen; and cortex, is part of an extensive functionally-organized network with the capacity to affect a broad range of basal ganglia functions (Smith et al. 2014).

The physiological contribution of the human thalamus to the network pathophysiology of GTS was elucidated in a longitudinal study which recorded changes in thalamic physiology over the course of DBS therapy (Maling et al. 2012). Local field potential (LFP) recordings from the CM/Pf revealed a correlation between tic symptomatology and spectral activity in the gamma band (see Fig. 6b). In patients responding to therapy, a clear correlation was shown between a decrease in tic severity and an increase in the power of synchronized gamma oscillations. This study demonstrated that, unlike other movement disorders in which the CBGTC is implicated, GTS dysfunction is a result of *hyposynchronization* in the thalamic network. The results of this investigation help elucidate the role of oscillations in GTS and offer insights into the basis of tic genesis and expression.

Alteration of the functional connectivity between regions within the CBGTC has also been described in individuals with GTS (Jeffries et al. 2002; Tinaz et al. 2014). Observations from ^18^F-fluorodeoxyglucose PET showed ventral striatum connectivity to be most prominent, although changes in the premotor areas, SMA, and insula were also been reported. Functional connections between the motor and lateral orbitofrontal circuits were the same in GTS patients and healthy controls, but the Tourette’s group showed a reverse polarity such that their activity was positively coupled (increased activity in one is correlated with increased activity in the other). These data are collected by indirect observations of metabolic coupling between brain regions. Electrophysiological measures of the directional neuronal connectivity in the network in humans have not been reported to date and would contribute to our understanding of the underlying functional mechanisms of the pathophysiology of GTS (Hashemiyoon et al. 2016).

## Treatment Options

There is presently no cure for Tourette syndrome. Furthermore, because almost all cases of GTS are co-occurring with neurobehavioral disorders, therapeutic options must aim at relieving tic severity and frequency in addition to treating the often more troublesome comorbid symptoms. As such, three features of GTS should be taken into consideration when planning ameliorative strategies: (1) individualized patient portrait, due to the variability of symptom profiles that arise from a range of comorbidities; (2) the waxing and waning of the location, frequency, and intensity of symptoms, which can occur over a range from minutes to months; and (3) the natural history of symptomatology, which increases then decreases from early childhood to adulthood. Treatment options are behavioral, chemical and/or surgical as symptom severity increases or as needed.

Due to new data on the efficacy of behavior therapy (especially “habit reversal training”), it is now recommended as a first-line intervention treatment for GTS (Verdellen et al. 2011; van de Griendt et al. 2013). While these therapies are becoming increasingly more favorably recognized as treatment options (in particular as safe and effective for children), results are comparatively slow onset and require further investigation to establish persistence of ameliorative effects. As side effects are not an issue with this option, it could be recommended as a supplemental add-on treatment, to be offered alongside other therapies.

Pharmacological interventions are typically introduced in moderate to severe cases. In such indications classic neuroleptics, which work as DA-antagonists, are still the most commonly prescribed because of their ability to relieve tic burden (Eddy et al. 2011). These drugs include pimozide and haloperidol, which are FDA-approved antipsychotics, or risperidone, which is first-option in Europe (Roessner et al. 2011). For milder cases—if treatment is recommended—α2-adrenergic agonists are indicated, in particular where ADHD comorbidity occurs (Robertson and Orth 2006; Singer 2010; McNaught and Mink 2011). For isolated motor tics, botulinum neurotoxin injections have been shown to be effective and well-tolerated (Kwak et al. 2000; Marras et al. 2001).

No tailored drug therapy has been developed for GTS; therefore, pharmacotherapy consists of remedies borrowed from other disorders, such as schizophrenia (McNaught and Mink 2011). The use of antipsychotics, while effective in alleviating motor symptoms, also gives rise to considerable side effects (McNaught and Mink 2011; Ganos and Martino 2015). This is due to their interaction with a range of neurotransmitter systems, including the histaminergic, serotonergic, cholinergic and alpha-adrenergic. This is a major complication of the available pharmacotherapy and thus compromises its practical effectiveness. The development of neuroleptics with high efficacy and low side effects, such as the now molecule-of-choice, Aripiprazole, would greatly improve the options for pharmaceutical intervention in GTS (Hartmann and Worbe 2013).

In medically refractory cases, where pharmacological intervention cannot provide relief, surgery is an option. Historically, surgical lesioning in a variety of target sites has been performed in debilitating cases. These included ablations in the thalamus, globus pallidus, frontal lobe (bimedial frontal leucotomy and prefrontal lobotomy), limbic system (anterior cingulotomy and limbic leucotomy), and cerebellum (Hassler and Dieckmann 1970; Temel and Visser-Vandewalle 2004; Singer 2010).

## DBS and Electrophysiology

### History

More recently, a safer surgical option, DBS, has been introduced (Vandewalle et al. 1999; Sassi et al. 2011). The procedure, which remains largely unchanged since its inception, requires the electrical stimulation of deep brain structures by chronically implanted electrodes which receive high-frequency pulses from an often subcutaneously implanted pulse generator.

DBS is FDA-approved and carries the CE-mark for movement disorders such as Parkinson’s disease and essential tremor and has been recently approved in a neuropsychiatric indication, OCD. Although it is not yet approved in the treatment of GTS, the procedure has been performed in over 120 patients in 25 centers across 14 countries since the first surgery in 1999 (Vandewalle et al. 1999; Müller-Vahl 2013; Schrock et al. 2014). The vast majority of the 48 studies published since then target only a single brain structure, with two targets in 10 cases and three targets in one case (Schrock et al. 2014). The first DBS surgery for GTS was performed in a 42 year-old male whose tics decreased from 38 per minute to zero at 12 months post-operatively (Vandewalle et al. 1999).

### Targeting: Surgical

The target described by Vandewalle et al. in this inaugural case was in the medial part of the thalamus (Vandewalle et al. 1999). The selection of this surgical focus was motivated by the thalamotomies described by Hassler in 1970 (Hassler and Dieckmann 1970; Temel and Visser-Vandewalle 2004; Singer 2010). The rationale for selection was to obtain the same clinical effect on GTS symptoms with the bilateral high frequency stimulation of DBS. Hassler performed between ten and fourteen coagulations in each hemisphere, in the medial, intralaminar and ventrolateral thalamic nuclei. In order to be able to stimulate the three groups of nuclei targeted by Hassler, the strategic point chosen was located at the intersection between the anteromedial border of the centre médian nucleus of the thalamus (CM), as part of the intralaminar thalamic nuclei; the periventricular substance (substania periventricularis (Spv)), as part of the medial thalamic nuclei; and the nucleus ventralis oralis internus (Voi), as part of the ventrolateral thalamic nuclei. The trajectory for the electrode is ideally along the longest axis of the Voi. Based on the Schaltenbrand-Wahren atlas, the coordinates for this target, in relation to the line connecting the anterior with the posterior commissure (AC–PC line), are: 5 mm lateral to the AC–PC line, 4 mm posterior to mid AC–PC line, and at the level of the AC–PC line (depth coordinate).

This target has been adopted by some centers, and slightly modified by others. For example, based on their positive clinical results, Servello et al. utilize a target which is 2 mm more anterior, while others stimulate the center of the CM (Welter et al. 2008; Maling et al. 2012; Servello et al. 2016).

Including the thalamus, target selection for DBS in GTS has totaled nine distinct brain regions, aligned with the functional neurobiopathology in the CBGTC circuit (Hariz and Robertson 2010; Sassi et al. 2011). Although the therapeutic potential of these targets is an area of active investigation, three brain regions are commonly implanted: (1) the medial thalamic region with 70 reported cases, (2) the GPi, with 30 cases reported (14 anteromedial (topographically limbic area), 16 posteroventral) and (3) the ALIC/NA with 6 reported cases (Schrock et al. 2014). Additionally, the GPe and the STN have each been reported once as a target, the latter in a Parkinson’s patient with tics. The most efficacious target appears to be the thalamus, but it is also the region most often targeted for surgical intervention.

### Targeting: Functional

In addition to the reasoning mentioned above, Vandewalle et al. based their choice of target on the hypothesis that the application of DBS to it would lead to the modulation of: (1) motor symptoms via stimulation of the CM, which projects to the motor striatum (motor feedback circuit); (2) behavioral symptoms via stimulation of the Spv, which projects to the ventral striatum (limbic circuit); and (3) facial tics (a predominant GTS symptom) via stimulation of the Voi, because of its direct projections to the facial part of the (pre-) motor cortex.

In the neurobiological dysfunction of GTS, the thalamus is a key component both anatomically and functionally (Haber and Calzavara 2009). It is intimately involved in both the processes (such as emotions, motivation and cognition) that lead to movement and the goal-directed behaviors that require movement. It is well connected to both the cortex and striatum. Located in the caudal part of the intralaminar thalamus, the CM/Pf diffusely innervates the motor, premotor and the prefrontal cortices (Parent and Parent 2005). Furthermore, in addition to being the target of integrated basal ganglia output, it projects dense clusters of axon terminals to focally innervate the striatum (Matsumoto et al. 2001). Beyond these, a third set of projection neurons jointly innervates both the cortex and the striatum (Parent and Parent 2005). These tightly connected functional circuits signify the importance of the CM/Pf, which has a pivotal role in the state setting modulatory system of the brain (where it affects both arousal and attention) and is intimately associated with the basal ganglia action-gating circuit (Matsumoto et al. 2001; Parent and Parent 2005).

The CM/Pf is well-situated to synthesize, organize and control the information transmitted to both the cortex and striatum. In other words, the parallel and ‘segregated’ pathways of the CBGTC create a rich network with the thalamus potentially “in the driver’s seat”, modulating other structures. The regulatory functions of the thalamus are already well-documented in other systems (e.g. visual and auditory). With the thalamus strategically positioned in this widely distributed, finely tuned, complex network, its use as a target for therapeutic DBS for GTS could resolve a more inclusive range of symptoms related to motor function as well as emotion and cognition.

Targeted stimulation in alternative deep brain areas should nonetheless be considered for highly circumscribed disease dimensions. Due to its various and high number of comorbidities, GTS presents with a broad phenotypic range. For patients with a stronger functional bias, other targets may be more therapeutically sensible for the relief of specific symptoms. For example, the posteroventrolateral part of the GPi could be selected for patients seeking to relieve robust motor symptoms, while the ALIC/NA would address neurobehavioral concerns such as obsessions and compulsions.

### Intra- and Peri-Operative Recordings

Despite a considerable body of work from imaging, pathological and other studies to suggest mechanisms for the pathophysiology of GTS, important questions still remain. DBS has offered the opportunity for acquiring direct evidence of the activity in one or more component regions of the suspected pathophysiological pathway from in vivo electrophysiological recordings in human patients. Intra-operative recordings from drivable microelectrodes present an opportunity for single unit recordings, while post-operative recordings of local field potentials can be made from implanted macroelectrodes. In 2010, the first thalamic physiological data from seven GTS patients undergoing DBS were reported from seven patients (Marceglia et al. 2010). Action potentials from single neurons in the trajectory targeting the Vo complex revealed irregular firing and oscillatory bursting at 4–6 mm and 1–3 mm above target respectively. At target, a quiet zone was reported although the authors suggest this may be due to the activity of the zona incerta which is actually below target.

Intra-operative single neuron recordings characterized bursting in the posterior Vo region of the thalamus in anaesthetized patients, consistent with low frequency thalamic bursting reported in the thalamic nuclei of various other neurological disorders (Marceglia et al. 2010; Priori et al. 2013). Analysis of local field potential (LFP) recordings in the same region in the same patients—while not ticcing—2 days post-operatively exhibited prominent oscillations in the delta, theta, and alpha range (Marceglia et al. 2010).

In eight patients undergoing pallidotomy, similar bursting activity, in addition to pauses in tonic activity, was observed in the GPi in 45 % of recorded neurons (Zhuang et al. 2009). Most of this activity was predominantly synchronous (~71 %). A little over 25 % of these occurred prior to EMG onset, mostly with increased activity (~78 %), although some exhibited decreased activity (22 %). A very small percentage of recorded neurons (~3 %) followed EMG onset. Pallidal neurons also showed decreased neuronal firing rate and irregular pattern.

### Acute and Chronic Recordings

Recordings from intra- and peri-operative studies offer a precious opportunity to extract information directly from the human brain in vivo. By themselves, however, they face severe limitations. While the thalamic study reported for the first time the electrophysiology of the Vo, the neurophysiological properties described were neither unique to the target nor to the disorder, GTS (Marceglia et al. 2010; Priori et al. 2013). Additionally, no other measures were taken to assure correlation to tic expression, and LFPs were taken in the absence of ticcing. The recordings of GPi neurons from patients undergoing pallidotomy addressed this issue by utilizing EMG to show correlation between neurophysiological behavior and tic behavior.

These studies provide first approaches to gaining a better understanding of the neurophysiology of relevant deep brain nuclei in the human GTS brain; however, such acute studies suffer from two major failings. First, they are potentially confounded by the notable microlesion effect, which can induce biochemical responses in neurons and persist up to 2–3 months post-operatively (Granziera et al. 2008; Pourfar et al. 2009; Tykocki et al. 2013). Second, they do not explain the underlying neurophysiological dynamics that support the pathology and how these are altered as a result of therapy. Such information must come from chronic recordings from in-dwelling electrodes.

Recordings from electrodes implanted inside the human brain offer a rare opportunity to observe neuronal behavior correlated with disease state. In 2012, Maling/Hashemiyoon et al. reported on the first and only study to date from direct, chronic recordings in the human CM thalamus over the course of DBS therapy (Maling et al. 2012). This investigation in a cohort of five subjects tracked over 6 months focused on the pathophysiological dynamics of the thalamic network in response to a therapeutic modality. Using the Neuropace™ system, LFP recordings were made from the implanted macroelectrodes of the DBS device and analyzed in relation to GTS symptomatology. Temporal analysis of the spectral characteristics of LFP recordings in the CM thalamus was correlated with the clinical benefit derived by each subject, typically tracked on a monthly basis. The results of this study showed the first clinical correlation between GTS symptomatology and a band-specific oscillatory frequency. A clear correlation was shown between a decrease in tic severity and an increase in the power of gamma synchronized oscillations. Patients with the best clinical outcomes over the duration of the study also exhibited the largest increases in gamma power at 6 months^2^ (see Fig. 6b). Non-responders to DBS did not show significant improvement clinically and did not exhibit gamma oscillations. Gamma band activity is characteristic of normal thalamic information processing (Llinas and Ribary 1993; Steriade et al. 1993, 1996). The therapeutic effect of DBS may be the functional restoration of effective communication. This is reflected in the increased synchronization of neurons contributing to gamma synchronized activity.

The short term effect of DBS on the thalamic network was also tested. One minute DBS-OFF recordings were taken during a relaxed awake state before and after 30 s stimulation at the therapeutic settings. Spectral difference plots showed a statistically significant increase in gamma power in the right hemisphere of three patients, two of them also responders in the chronic DBS data set (see Fig. 6c). No significant changes were seen in any of the patients’ left hemispheres.

As the first ever longitudinal study of the effects of DBS on the dynamic changes in specific frequency bands in human GTS patients, this study addresses a vital gap in our knowledge of the neural correlates of symptomatology as well as dynamic physiological responses to DBS therapy over time. The results of this study suggest that dynamic changes in gamma band power are relevant to the pathophysiology underlying GTS, and support the hypothesis that the CM thalamus is an important therapeutic focus in its pathological circuit dysfunction.

### Network Dynamics

The functional relevance of the thalamic network is suggested by both the gamma amplitude and its correlation strength to improved symptomatology when comparing results from more circumscribed bipolar electrode recordings to that of the largest sampling area. Although all channels showed changes in gamma power correlated with decreased tic severity, the fluctuations were greatest in channel four. This channel represented the largest sampling of population activity because it had the largest spatial sampling area (the greatest possible lead distribution, across contacts 1–4) (see Fig. 6a).

The collective activity of neuronal ensembles gives rise to neuronal oscillations, which reflect the specific temporal coordination of the population. There is extensive evidence that this coordinated activity (synchronized oscillations) is associated with a wide range of functions as part of the normal communication found in healthy brain processes. Pathological states of brain dysfunction are expressed as altered coordination states, represented neurophysiologically as perverted rhythms. Aberrant oscillations have been associated with a variety of disorders and have become the focus of intense examination over the last years (Llinas et al. 1999; Brown 2003; Uhlhaas and Singer 2006; Maling et al. 2012).

One theory which sparked the movement towards understanding neurological and neuropsychiatric disorders in terms of abnormal synchronization patterns is that of thalamocortical dysrhythmia (TCD) (Llinas et al. 1999; McCormick 1999). TCD suggests that the mechanism that produces disorders from Parkinson’s disease to depression is the same and that it is functionally based on the electrophysiological properties of the thalamocortical loop. The two main tenants of TCD are (1) a shift to persistent theta oscillations and (2) large-scale coherence that unremittingly restricts and alters the dynamic reorganization of the brain. This widespread activity results in an ectopic gamma that arises from “an edge effect”.

So influential was this theory that it was purported to be the mechanism underlying tic genesis in GTS by one of the most renowned figures in the field (Leckman et al. 2006). In the absence of any direct, electrophysiological recordings to the contrary, this hypothesis of hypersynchronization was entirely plausible. In order to test the theory and definitively determine its validity, similar analyses were performed using the chronic LFP recordings of the thalamus.

The first line of comparison was a determination of theta behavior in GTS. To adhere to the most important characteristic distinguishing theta rhythmicity, theta power would have to be either consistently high, or increase with increased severity of symptoms. Neither was the case. In fact no consistent correlation was observed between the theta band and changes in symptomatology, its behavior seemed independent of such changes (Maling et al. 2012).

The second and perhaps most telling analysis was of spectral power correlation plots. Using the nearly identical algorithm described in the original paper, frequencies were plotted to find the cross-frequency coherence between theta and gamma power. Here the results were quite interesting. Increased theta-gamma correlations were not linked to greater dysfunctional state, but rather the contrary (unpublished results). They were observed to be *increasing* in coherence in correlation with *improving* symptomatology. However, similar to fluctuations purely in the gamma band, the results were non-linear. Rather, the largest power changes occurred when the greatest change in tic severity occurred as indicated by a decrease inYGTSS scores. Thus, it wasn’t the lowest score that showed the highest coherence, but rather the greatest *drop* in score (∆).

Additionally, dynamic patterning was observed in the coherence matrix between frequencies which fluctuated with behavioral state (personal observation). These alterations in the coordination matrices of recorded ensembles are a reflection of network activity and thus, some functional parameter of information processing in the thalamus. As there is a close correlation between aberrant oscillatory activity and variation in the disease state (e.g. Parkinson’s disease (aberrant beta) versus GTS (aberrant gamma)), dynamic changes that occur in neuronal networks in response to DBS therapy contribute specific information to the possible pathological mechanism of impairment among other things in the coherence matrix. The results of this study, therefore, suggest that oscillations have a functional role in the pathophysiology of GTS.

## Conclusions, Discussion and Future Directions

There is at present no scientifically described category for Gilles de la Tourette syndrome (Cavanna and Rickards 2013). While there is much contributory information accounting for it, there is an enormous explanatory gap between the descriptive definition presently available and a true neurobiological understanding of it. Like other psychiatric disorders, diagnosis is “restricted to subjective symptoms and observable signs” (Insel and Cuthbert 2015). The rigorous application of behavioral, biological and computational measures to the dysfunctional brain states giving rise to GTS and other psychiatric disorders will not only offer a more complete characterization of them, but also provide firm, scientifically quantifiable criterion for them. This lays the foundation for precision medicine, where distinct aspects of underlying dysfunction can be targeted to deliver consistently successful therapies for optimized patient care.

Because definitive diagnostic tests are as of yet still lacking, GTS is presently classified by the observation of symptoms. As comorbidities can be exceedingly high (up to 90 % in specialized clinics), in particular with OCD and ADHD, it becomes nontrivial to establish the etiological basis of the wide range of phenotypes observed in such patients (Grados and Mathews 2009). The complex profile regularly—and rightfully—attributed to these individuals has led to the practical consideration of typing GTS. When the typing includes OCD, three classes emerge: TS only, TS + OCD, and TS + OCD + ADHD. Of these, the first and the last are considered authentically heritable. This is particularly interesting—and confounding—when one considers how intimately compulsions are related to tics; and yet, it is the addition of ADHD that provides evidence of a shared genetic foundation.

It has been suggested that GTS is a neuropsychiatric spectrum disorder. While it shares many commonalities with OCD and ADHD—and all three are disorders exhibiting excessive activity—the strong asymmetries between their profiles are a red flag. Of particular note is the extremely low comorbidity of OCD/ADHD with GTS. This small percentage may represent where the true heritability between these groups can be found, substantiating the conclusions of the GWAS of GTS and OCD: that these highly comorbid disorders have a complex genetic relationship but do not share a genetic architecture. This could be expressed as similarities and differences in pathophysiological mechanisms and neural networks circuits. The correlations and asymmetries observed in GTS comorbidities could be a staple for gene studies which go beyond the simple phenotypic expression of observable traits.

Exploration of the genetic basis of certain endophenotypes may be profitable (Gould and Gottesman 2006). GTS presents with a broad range of phenotypes which may arise from a heterogeneous genetic profile. Plausibly, the translation of such varied genetic expression is the variety of structural and functional abnormalities detected in the Tourette brain. Such a complex clinical and biological picture is especially challenging for the discovery of risk-conferring common genetic variants. It is unsurprising then, that GWAS have thus far not reported any. A promising method for addressing these issues is gene network analysis. Analyses based on differential gene expression with gene co-expression allow the identification of relevant gene networks of the disease state to be distinguished. By examining co-regulated patterns of gene expression, gene network analysis can link the translational changes in the expression of gene clusters with clinical reports of the observed endophenotypes.

GTS has been studied extensively using imaging and other indirect techniques that have shown structural and functional abnormalities in the contributors to the CBGTC circuit. However, results of these studies are conflicting and/or have not been replicated, in addition to being inherently more circumstantial. Moving forward, these studies would benefit from increased parameter control, which includes behavioral states, therapeutic effects (such as neuroleptics), and comorbidities.

Individuals with GTS necessarily suffer from abnormal brain organization. As a self-limiting neurodevelopmental disorder, examination of the alterations in the connectivity dynamics of the CBGTC over the natural history of the disorder would reveal the dynamic spatiotemporal equilibrium states of the network correlating with its dictated emergent behavioral state. Perturbations in the balance of the anatomical and functional connections in the circuits of this ever-important, delicately-poised yet robust network manifest as the cognitive, motor, and/or limbic deficits in dysfunctional disease states. Fluctuations in the coordination matrices of the CBGTC network likely actualize as the biological basis of a wide range of behaviors associated with the signs and symptoms of various disorders including Parkinson’s disease, Huntington’s disease, GTS, OCD, ADHD and others.

Intrinsic or extrinsic mechanisms, like plasticity or DBS, may be employed to recalibrate the neuronal equilibrium of network interactions (McIntyre et al. 2004; Babadi and Abbott 2010; Maling et al. 2012; Hashemiyoon 2013). This could be quantitatively measured by analysis of the changes in the activation of neuronal ensembles, such as indicated by alteration in their levels of synchronization. Such calculations directly address important questions about the underlying mechanisms of GTS pathophysiology.

Aberrant oscillatory behavior has been suggested to subserve various neuropsychiatric disorders and has been reported in GTS (Llinas et al. 1999; Uhlhaas and Singer 2006; Maling et al. 2012). Changes in the synchronized rhythms of specific frequency band activity (such as gamma) are reflected as neurophysiological fluctuations that could be correlated with alterations in motor and/or behavioral impairment, measurable by a relevant clinical metric (such as the YGTSS). DBS offers the unique opportunity to take direct electrophysiological recordings from the purported areas of dysfunction in neuropsychiatric disorders in humans. By tracking specific neurobiological changes in dysfunctional networks, potential biomarkers for GTS could be revealed.

Unfortunately, there are precious few direct investigations of the dynamic changes in pathology in humans. To date, there is only one longitudinal study reported in human subjects to quantify the underlying neurophysiological dynamics as correlated with therapeutic effect (Maling et al. 2012). That study revealed changes in the synchronization of gamma oscillations in the thalamic network of GTS patients in correlation with changes in symptomatology resulting from DBS therapy. Furthermore, changes in theta/gamma coherence have been correlated with changes in therapeutic benefit. The discovery of these neuromarkers is non-trivial, both scientifically and clinically.

Linking observations from different methodologies could provide some requisite quantifiable results. Demonstrated here for the first time is a mechanism by which alteration in the genetic domain generates a neuropathophysiological modification of network dynamics correlated with tic expression in GTS. Combining results from genetic network analyses revealing changes in interneuron-related genes with results from the longitudinal DBS study indicating changes in oscillatory synchronization illustrates a translational effect from molecular mechanisms to network neurophysiology which correlates with tic symptomatology. The down-regulation of hundreds of interneuron-related genes affects the balance of excitation/inhibition necessary for proper information processing in the brain. This imbalance in turn alters the structural and functional organization of the ensembles giving rise to synchronized oscillatory behavior. Such changes are expressed as an increase or decrease in the coordinated activity of neuronal ensembles which manifests as hyper- or hypo-synchronized oscillations. Thus, results from a genetic study substantiate the findings from a DBS investigation concluding that hyposynchronization of oscillations are correlated with tic expression. These combined approaches offer powerful insights into tic genesis in GTS and the functional role of oscillations in its dysfunction.

Studies in human subjects are especially important for psychiatric disorders, such as GTS, which suffer from insufficient animal models (Salgado and Sandner 2013). Results from human investigations can be apparently and swiftly applicable to the understanding and treatment of the associated disorders, and effect the development of improved therapeutic interventions.

Knowledge of biomarkers and their specific characteristic involvement in the disease state are valuable for the development of next-generation technologies such as closed-loop stimulation. Patient care would greatly benefit because of: (1) on-board improvements in target localization intra-operatively, (2) tailored treatment postoperatively, and (3) objective therapeutic assessment of the efficacy of stimulation parameters.

Further improvement of therapeutic strategies for patient care will come from studies which expand their scope. In order to elucidate the role of the various structures in the complex network giving rise to GTS, simultaneous recordings from subcortical networks concomitant with cortical recordings are needed. The inclusion of modern electrical neuroimaging methods with DBS studies is pivotal to a more comprehensive explanation of the mechanisms subserving GTS (Michel and Murray 2012; Hashemiyoon et al. 2016). Further investigations into oscillations and their aberrations in the larger networks of the CBGTC and brain are essential for understanding causality, function and the regulation of information integration. Investigation of these networks on a patient-by-patient basis using combined cortical-subcortical recordings delivers an improved estimation of the communication between the circuit nodes for each individual and provides a platform for personalized medicine.

## Footnotes

1 Although the term ‘vocal tic’ is used extensively, some tics, such as sniffing, do not engage the vocal chords; thus, the term phonic tic is a more accurate representation of the phenomenon.

2 One patient was presented at 5 months.

## References

[CR1] Abelson JF, Kwan KY, O’Roak BJ (2005). Sequence variants in SLITRK1 are associated with Tourette’s syndrome. Science.

[CR2] Albin RL, Mink JW (2006). Recent advances in Tourette syndrome research. Trends Neurosci.

[CR3] Alexander GE, DeLong MR, Strick PL (1986). Parallel organization of functionally segregated circuits linking basal ganglia and cortex. Annu Rev Neurosci.

[CR4] Ali F, Morrison KE, Cavanna AE (2013). The complex genetics of Gilles de la Tourette syndrome: implications for clinical practice. Neuropsychiatry.

[CR5] American Psychiatric Association (2013). Diagnostic and statistical manual of mental disorders.

[CR6] Aron AR (2011). From reactive to proactive and selective control: developing a richer model for stopping inappropriate responses. Biol Psychiatry.

[CR7] Aylward EH, Reiss AL, Reader MJ, Singer HS, Brown JE, Denckla MB (1996). Basal ganglia volumes in children with attention-deficit hyperactivity disorder. J Child Neurol.

[CR8] Babadi B, Abbott LF (2010). Intrinsic stability of temporally shifted spike-timing dependent plasticity. PLoS Comput Biol.

[CR9] Bar-Gad I, Bergman H (2001). Stepping out of the box: information processing in the neural networks of the basal ganglia. Curr Opin Neurobiol.

[CR10] Barkley R (1993). Attention-deficit hyperactivity disorder: a handbook for diagnosis and treatment.

[CR11] Baron-Cohen S, Scahill VL, Izaguirre J, Hornsey H, Robertson MM (1999). The prevalence of Gilles de la Tourette syndrome in children and adolescents with autism: a large scale study. Psychol Med.

[CR12] Bloch MH, Leckman JF (2009). Clinical course of Tourette syndrome. J Psychosom Res.

[CR13] Bloch MH, Leckman JF, Zhu H, Peterson BS (2005). Caudate volumes in childhood predict symptom severity in adults with Tourette syndrome. Neurology.

[CR14] Bohlhalter S, Goldfine A, Matteson S (2006). Neural correlates of tic generation in Tourette syndrome: an event-related functional MRI study. Brain.

[CR15] Brown P (2003). Oscillatory nature of human basal ganglia activity: relationship to the pathophysiology of Parkinson’s disease. Mov Disord.

[CR16] Burd L, Freeman RD, Klug MG, Kerbeshian J (2005). Tourette syndrome and learning disabilities. BMC Pediatr.

[CR17] Buse J, Schoenefeld K, Munchau A, Roessner V (2013). Neuromodulation in Tourette syndrome: dopamine and beyond. Neurosci Biobehav Rev.

[CR18] Capriotti MR, Himle MB, Woods DW (2014). Behavioral treatments for Tourette syndrome. J Obs Compuls Relat Disord.

[CR19] Castellan Baldan L, Williams KA, Gallezot JD (2014). Histidine decarboxylase deficiency causes tourette syndrome: parallel findings in humans and mice. Neuron.

[CR20] Cavanna AE, Rickards H (2013). The psychopathological spectrum of Gilles de la Tourette syndrome. Neurosci Biobehav Rev.

[CR21] Cavanna AE, Cavanna S, Monaco F (2008). Anger symptoms and “delinquent” behavior in Tourette syndrome with and without attention deficit hyperactivity disorder. Brain Dev.

[CR22] Chase TN, Foster NL, Fedio P, Brooks R, Mansi L, Kessler R, Di Chiro G (1984). Gilles de la tourette syndrome: studies with the fluorine-18-labeled fluorodeoxyglucose positron emission tomographic method. Ann Neurol.

[CR23] Coffey BJ, Biederman J, Smoller JW, Geller DA, Sarin P, Schwartz S, Kim GS (2000). Anxiety disorders and tic severity in juveniles with Tourette’s disorder. J Am Acad Child Adolesc Psychiatry.

[CR24] Cohen AJ, Leckman JF (1992). Sensory phenomena associated with Gilles de la Tourette’s syndrome. J Clin Psychiatry.

[CR25] Cohen SC, Leckman JF, Bloch MH (2013). Clinical assessment of Tourette syndrome and tic disorders. Neurosci Biobehav Rev.

[CR26] Conelea CA, Woods DW (2008). The influence of contextual factors on tic expression in Tourette’s syndrome: a review. J Psychosom Res.

[CR27] Cui Y, Jin Z, Chen X, He Y, Liang X, Zheng Y (2014). Abnormal baseline brain activity in drug-naive patients with Tourette syndrome: a resting-state fMRI study. Front Hum Neurosci.

[CR28] Denys D, Tenney N, van Megen HJ, de Geus F, Westenberg HG (2004). Axis I and II comorbidity in a large sample of patients with obsessive–compulsive disorder. J Affect Disord.

[CR29] Doyon J, Bellec P, Amsel R (2009). Contributions of the basal ganglia and functionally related brain structures to motor learning. Behav Brain Res.

[CR30] Draganski B, Martino D, Cavanna AE (2010). Multispectral brain morphometry in Tourette syndrome persisting into adulthood. Brain.

[CR31] Draper A, Stephenson MC, Jackson GM, Pepes S, Morgan PS, Morris PG, Jackson SR (2014). Increased GABA contributes to enhanced control over motor excitability in Tourette syndrome. Curr Biol.

[CR32] Eapen V, Robertson MM (2015). Are there distinct subtypes in Tourette syndrome? Pure-Tourette syndrome versus Tourette syndrome-plus, and simple versus complex tics. Neuropsychiatr Dis Treat.

[CR33] Eddy CM, Rickards HE, Cavanna AE (2011). Treatment strategies for tics in Tourette syndrome. Ther Adv Neurol Disord.

[CR34] Ercan-Sencicek AG, Stillman AA, Ghosh AK (2010). L-histidine decarboxylase and Tourette’s syndrome. N Engl J Med.

[CR35] Fahn S, Jankovic J, Hallett M, Fahn S, Jankovic J, Hallett M (2011). Tics and Tourette syndrome. Principles and practice of movement disorders.

[CR36] Faraone SV, Sergeant J, Gillberg C, Biederman J (2003). The worldwide prevalence of ADHD: is it an American condition?. World Psychiatry.

[CR37] Felling RJ, Singer HS (2011). Neurobiology of tourette syndrome: current status and need for further investigation. J Neurosci.

[CR38] Flaherty AW, Graybiel AM (1991). Corticostriatal transformations in the primate somatosensory system. Projections from physiologically mapped body-part representations. J Neurophysiol.

[CR39] Frank MJ (2009). Slave to the striatal habit (Commentary on Tricomi et al.). Eur J Neurosci.

[CR40] Freeman RD (2007). Tic disorders and ADHD: answers from a world-wide clinical dataset on Tourette syndrome. Eur Child Adolesc Psychiatry.

[CR41] Freeman RD, Fast DK, Burd L, Kerbeshian J, Robertson MM, Sandor P (2000). An international perspective on Tourette syndrome: selected findings from 3500 individuals in 22 countries. Dev Med Child Neurol.

[CR42] Freeman RD, Zinner SH, Muller-Vahl KR (2009). Coprophenomena in Tourette syndrome. Dev Med Child Neurol.

[CR43] Fried I, Katz A, McCarthy G, Sass KJ, Williamson P, Spencer SS, Spencer DD (1991). Functional organization of human supplementary motor cortex studied by electrical stimulation. J Neurosci.

[CR44] Ganos C, Martino D (2015). Tics and tourette syndrome. Neurol Clin.

[CR45] Ganos C, Asmuss L, Bongert J, Brandt V, Munchau A, Haggard P (2015). Volitional action as perceptual detection: predictors of conscious intention in adolescents with tic disorders. Cortex.

[CR46] Ganos C, Garrido A, Navalpotro-Gomez I (2015). Premonitory urge to tic in Tourette’s is associated with interoceptive awareness. Mov Disord.

[CR47] Ghanizadeh A, Mosallaei S (2009). Psychiatric disorders and behavioral problems in children and adolescents with Tourette syndrome. Brain Dev.

[CR48] Goetz CG, Tanner CM, Stebbins GT, Leipzig G, Carr WC (1992). Adult tics in Gilles de la Tourette’s syndrome: description and risk factors. Neurology.

[CR49] Goldenberg JN, Brown SB, Weiner WJ (1994). Coprolalia in younger patients with Gilles de la Tourette syndrome. Mov Disord.

[CR50] Gould TD, Gottesman II (2006). Psychiatric endophenotypes and the development of valid animal models. Genes Brain Behav.

[CR51] Grados MA, Mathews CA (2008). Latent class analysis of Gilles de la Tourette syndrome using comorbidities: clinical and genetic implications. Biol Psychiatry.

[CR52] Grados MA, Mathews CA (2009). Clinical phenomenology and phenotype variability in Tourette syndrome. J Psychosom Res.

[CR53] Granziera C, Pollo C, Russmann H (2008). Sub-acute delayed failure of subthalamic DBS in Parkinson’s disease: the role of micro-lesion effect. Parkinsonism Relat Disord.

[CR54] Graybiel AM (2000). The basal ganglia. Curr Biol.

[CR55] Graybiel AM (2005). The basal ganglia: learning new tricks and loving it. Curr Opin Neurobiol.

[CR56] Haber SN, Tarsy D, Vitek JL, Starr PA, Okun MS (2008). Functional anatomy and physiology of the basal ganglia: non-motor functions. Current clinical neurology: deep brain stimulation in neurological and psychiatric disorders.

[CR57] Haber SN, Calzavara R (2009). The cortico-basal ganglia integrative network: the role of the thalamus. Brain Res Bull.

[CR58] Hallett M, Cohen DJ, Jankovic J, Goetz CG (2001). Neurophysiology of tics. Tourette syndrome. Advances in neurology.

[CR59] Hallett M (2015). Tourette syndrome: update. Brain Dev.

[CR60] Hammond C, Bergman H, Brown P (2007). Pathological synchronization in Parkinson’s disease: networks, models and treatments. Trends Neurosci.

[CR61] Hampson M, Tokoglu F, King RA, Constable RT, Leckman JF (2009). Brain areas coactivating with motor cortex during chronic motor tics and intentional movements. Biol Psychiatry.

[CR62] Hanna PA, Janjua FN, Contant CF, Jankovic J (1999). Bilineal transmission in Tourette syndrome. Neurology.

[CR63] Hariz MI, Robertson MM (2010). Gilles de la Tourette syndrome and deep brain stimulation. Eur J Neurosci.

[CR64] Hartmann A, Worbe Y (2013). Pharmacological treatment of Gilles de la Tourette syndrome. Neurosci Biobehav Rev.

[CR65] Hashemiyoon R (2013) Plasticity, neuronal synchrony and multi-stability in the human thalamus. In: Proceedings of the twenty second annual computational neuroscience meeting, Paris, France. doi: 10.13140/2.1.4707.4249

[CR66] Hashemiyoon R, Tomescu M, Coito A (2016). Effective connectivity of subcortical-cortical networks revealed by simultaneous scalp and depth EEG recordings in humans. Clin Neurophysiol.

[CR67] Hassler R, Dieckmann G (1970). Stereotaxic treatment of tics and inarticulate cries or coprolalia considered as motor obsessional phenomena in Gilles de la Tourette’s disease. Rev Neurol.

[CR68] Hazrati LN, Parent A (1992). Convergence of subthalamic and striatal efferents at pallidal level in primates: an anterograde double-labeling study with biocytin and PHA-L. Brain Res.

[CR69] Hazrati LN, Parent A (1992). Differential patterns of arborization of striatal and subthalamic fibers in the two pallidal segments in primates. Brain Res.

[CR70] Hyde TM, Aaronson BA, Randolph C, Rickler KC, Weinberger DR (1992). Relationship of birth weight to the phenotypic expression of Gilles de la Tourette’s syndrome in monozygotic twins. Neurology.

[CR71] Hyde TM, Stacey ME, Coppola R, Handel SF, Rickler KC, Weinberger DR (1995). Cerebral morphometric abnormalities in Tourette’s syndrome: a quantitative MRI study of monozygotic twins. Neurology.

[CR72] Insel TR, Cuthbert BN (2015). Medicine. Brain disorders? Precisely. Science.

[CR73] Israelashvili M, Loewenstern Y, Bar-Gad I (2015). Abnormal neuronal activity in Tourette syndrome and its modulation using deep brain stimulation. J Neurophysiol.

[CR74] Jahanshahi M, Obeso I, Rothwell JC, Obeso JA (2015). A fronto-striato-subthalamic-pallidal network for goal-directed and habitual inhibition. Nat Rev Neurosci.

[CR75] Jankovic J (1997). Tourette syndrome. Phenomenology and classification of tics. Neurol Clin.

[CR76] Jankovic J (2001). Tourette’s syndrome. N Engl J Med.

[CR77] Jankovic J, Stone L (1991). Dystonic tics in patients with Tourette’s syndrome. Mov Disord.

[CR78] Jeffries KJ, Schooler C, Schoenbach C, Herscovitch P, Chase TN, Braun AR (2002). The functional neuroanatomy of Tourette’s syndrome: an FDG PET study III: functional coupling of regional cerebral metabolic rates. Neuropsychopharmacology.

[CR79] Joel D, Weiner I (1994). The organization of the basal ganglia–thalamocortical circuits: open interconnected rather than closed segregated. Neuroscience.

[CR80] Kadesjo B, Gillberg C (2000). Tourette’s disorder: epidemiology and comorbidity in primary school children. J Am Acad Child Adolesc Psychiatry.

[CR81] Kalanithi PS, Zheng W, Kataoka Y (2005). Altered parvalbumin-positive neuron distribution in basal ganglia of individuals with Tourette syndrome. Proc Natl Acad Sci USA.

[CR82] Karagiannidis I, Dehning S, Sandor P (2013). Support of the histaminergic hypothesis in Tourette syndrome: association of the histamine decarboxylase gene in a large sample of families. J Med Genet.

[CR83] Kataoka Y, Kalanithi PS, Grantz H, Schwartz ML, Saper C, Leckman JF, Vaccarino FM (2010). Decreased number of parvalbumin and cholinergic interneurons in the striatum of individuals with Tourette syndrome. J Comp Neurol.

[CR84] Kornhuber HH, Deecke L (1965). Changes in the brain potential in voluntary movements and passive movements in man: readiness potential and reafferent potentials. Pflugers Arch.

[CR85] Kurlan R, Como PG, Miller B (2002). The behavioral spectrum of tic disorders: a community-based study. Neurology.

[CR86] Kwak CH, Hanna PA, Jankovic J (2000). Botulinum toxin in the treatment of tics. Arch Neurol.

[CR87] Kwak C, Vuong KD, Jankovic J (2003). Migraine headache in patients with Tourette syndrome. Arch Neurol.

[CR88] Lai CH, Wu YT (2015). The patterns of fractional amplitude of low-frequency fluctuations in depression patients: the dissociation between temporal regions and fronto-parietal regions. J Affect Disord.

[CR89] Leckman JF (2002). Tourette’s syndrome. Lancet.

[CR90] Leckman JF (2003). Phenomenology of tics and natural history of tic disorders. Brain Dev.

[CR91] Leckman JF, Walker DE, Cohen DJ (1993). Premonitory urges in Tourette’s syndrome. Am J Psychiatry.

[CR92] Leckman JF, Peterson BS, Anderson GM, Arnsten AF, Pauls DL, Cohen DJ (1997). Pathogenesis of Tourette’s syndrome. J Child Psychol Psychiatry.

[CR93] Leckman JF, Vaccarino FM, Kalanithi PS, Rothenberger A (2006). Annotation: tourette syndrome: a relentless drumbeat—driven by misguided brain oscillations. J Child Psychol Psychiatry.

[CR94] Leckman JF, Bloch MH, Smith ME, Larabi D, Hampson M (2010). Neurobiological substrates of Tourette’s disorder. J Child Adolesc Psychopharmacol.

[CR95] Leisman G, Braun-Benjamin O, Melillo R (2014). Cognitive-motor interactions of the basal ganglia in development. Front Syst Neurosci.

[CR96] Lenglet C, Abosch A, Yacoub E, De Martino F, Sapiro G, Harel N (2012). Comprehensive in vivo mapping of the human basal ganglia and thalamic connectome in individuals using 7T MRI. PLoS ONE.

[CR97] Lennington JB, Coppola G, Kataoka-Sasaki Y (2016). Transcriptome analysis of the human striatum in Tourette syndrome. Biol Psychiatry.

[CR98] Libet B (1985). Unconscious cerebral initiative and the role of conscious will in voluntary action. Behav Brain Sci.

[CR99] Liu Y, Miao W, Wang J (2013). Structural abnormalities in early Tourette syndrome children: a combined voxel-based morphometry and tract-based spatial statistics study. PLoS ONE.

[CR100] Llinas R, Ribary U (1993). Coherent 40-Hz oscillation characterizes dream state in humans. Proc Natl Acad Sci USA.

[CR101] Llinas RR, Ribary U, Jeanmonod D, Kronberg E, Mitra PP (1999). Thalamocortical dysrhythmia: a neurological and neuropsychiatric syndrome characterized by magnetoencephalography. Proc Natl Acad Sci USA.

[CR102] Maia TV (2009). Reinforcement learning, conditioning, and the brain: successes and challenges. Cogn Affect Behav Neurosci.

[CR103] Maia TV, Frank MJ (2011). From reinforcement learning models to psychiatric and neurological disorders. Nat Neurosci.

[CR104] Maling N, Hashemiyoon R, Foote KD, Okun MS, Sanchez JC (2012). Increased thalamic gamma band activity correlates with symptom relief following deep brain stimulation in humans with Tourette’s syndrome. PLoS ONE.

[CR105] Marceglia S, Servello D, Foffani G (2010). Thalamic single-unit and local field potential activity in Tourette syndrome. Mov Disord.

[CR106] Marras C, Andrews D, Sime E, Lang AE (2001). Botulinum toxin for simple motor tics: a randomized, double-blind, controlled clinical trial. Neurology.

[CR107] Marsh R, Alexander GM, Packard MG, Zhu H, Wingard JC, Quackenbush G, Peterson BS (2004). Habit learning in Tourette syndrome: a translational neuroscience approach to a developmental psychopathology. Arch Gen Psychiatry.

[CR108] Marsh R, Zhu H, Wang Z, Skudlarski P, Peterson BS (2007). A developmental fMRI study of self-regulatory control in Tourette’s syndrome. Am J Psychiatry.

[CR109] Matsumoto N, Minamimoto T, Graybiel AM, Kimura M (2001). Neurons in the thalamic CM-Pf complex supply striatal neurons with information about behaviorally significant sensory events. J Neurophysiol.

[CR110] McCairn KW, Nagai Y, Hori Y (2016). A primary role for nucleus accumbens and related limbic network in vocal tics. Neuron.

[CR111] McCormick DA (1999). Are thalamocortical rhythms the Rosetta Stone of a subset of neurological disorders?. Nat Med.

[CR112] McIntyre CC, Savasta M, Kerkerian-Le Goff L, Vitek JL (2004). Uncovering the mechanism(s) of action of deep brain stimulation: activation, inhibition, or both. Clin Neurophysiol.

[CR113] McNaught KS, Mink JW (2011). Advances in understanding and treatment of Tourette syndrome. Nat Rev Neurol.

[CR114] Meidinger AL, Miltenberger RG, Himle M, Omvig M, Trainor C, Crosby R (2005). An investigation of tic suppression and the rebound effect in Tourette’s disorder. Behav Modif.

[CR115] Melillo R, Leisman G (2009). Neurobehavioral disorders of childhood: an evolutionary perspective.

[CR116] Michel CM, Murray MM (2012). Towards the utilization of EEG as a brain imaging tool. Neuroimage.

[CR117] Mink JW (2001). Basal ganglia dysfunction in Tourette’s syndrome: a new hypothesis. Pediatr Neurol.

[CR118] Mohammadi MR, Ghanizadeh A, Rahgozar M (2004). Prevalence of obsessive–compulsive disorder in Iran. BMC Psychiatry.

[CR119] Moustafa AA, Bar-Gad I, Korngreen A, Bergman H (2014). Basal ganglia: physiological, behavioral, and computational studies. Front Syst Neurosci.

[CR120] Müller-Vahl KR (2013). Surgical treatment of Tourette syndrome. Neurosci Biobehav Rev.

[CR121] Nambu A (2007). Globus pallidus internal segment. Prog Brain Res.

[CR122] Nambu A (2008). Seven problems on the basal ganglia. Curr Opin Neurobiol.

[CR123] Nambu A, Itakura T (2015). Functional circuitry of the basal ganglia. DBS for neurological disorders.

[CR124] Nambu A, Tokuno H, Takada M (2002). Functional significance of the cortico–subthalamo–pallidal ‘hyperdirect’ pathway. Neurosci Res.

[CR125] Obeso JA, Rothwell JC, Marsden CD (1981). Simple tics in Gilles de la Tourette’s syndrome are not prefaced by a normal premovement EEG potential. J Neurol Neurosurg Psychiatry.

[CR126] O’Connor K, Brisebois H, Brault M, Robillard S, Loiselle J (2003). Behavioral activity associated with onset in chronic tic and habit disorder. Behav Res Ther.

[CR127] Papa SM, Artieda J, Obeso JA (1991). Cortical activity preceding self-initiated and externally triggered voluntary movement. Mov Disord.

[CR128] Parent M, Parent A (2005). Single-axon tracing and three-dimensional reconstruction of centre median-parafascicular thalamic neurons in primates. J Comp Neurol.

[CR129] Pauls DL, Leckman JF (1986). The inheritance of Gilles de la Tourette’s syndrome and associated behaviors. Evidence for autosomal dominant transmission. N Engl J Med.

[CR130] Peterson BS, Leckman JF (1998). The temporal dynamics of tics in Gilles de la Tourette syndrome. Biol Psychiatry.

[CR131] Peterson BS, Staib L, Scahill L (2001). Regional brain and ventricular volumes in Tourette syndrome. Arch Gen Psychiatry.

[CR132] Peterson BS, Thomas P, Kane MJ (2003). Basal Ganglia volumes in patients with Gilles de la Tourette syndrome. Arch Gen Psychiatry.

[CR133] Piacentini J, Himle MB, Chang S, Baruch DE, Buzzella BA, Pearlman A, Woods DW (2006). Reactivity of tic observation procedures to situation and setting. J Abnorm Child Psychol.

[CR134] Piacentini J, Woods DW, Scahill L (2010). Behavior therapy for children with Tourette disorder: a randomized controlled trial. JAMA.

[CR135] Pittenger C, Bloch MH, Williams K (2011). Glutamate abnormalities in obsessive compulsive disorder: neurobiology, pathophysiology, and treatment. Pharmacol Ther.

[CR136] Plessen KJ, Wentzel-Larsen T, Hugdahl K (2004). Altered interhemispheric connectivity in individuals with Tourette’s disorder. Am J Psychiatry.

[CR137] Plessen KJ, Bansal R, Peterson BS (2009). Imaging evidence for anatomical disturbances and neuroplastic compensation in persons with Tourette syndrome. J Psychosom Res.

[CR138] Porta M, Brambilla A, Cavanna AE, Servello D, Sassi M, Rickards H, Robertson MM (2009). Thalamic deep brain stimulation for treatment-refractory Tourette syndrome: two-year outcome. Neurology.

[CR139] Porta M, Servello D, Sassi M, Brambilla A, Defendi S, Priori A, Robertson M (2009). Issues related to deep brain stimulation for treatment-refractory Tourette’s syndrome. Eur Neurol.

[CR140] Pourfar M, Tang C, Lin T, Dhawan V, Kaplitt MG, Eidelberg D (2009). Assessing the microlesion effect of subthalamic deep brain stimulation surgery with FDG PET. J Neurosurg.

[CR141] Price RA, Kidd KK, Cohen DJ, Pauls DL, Leckman JF (1985). A twin study of Tourette syndrome. Arch Gen Psychiatry.

[CR142] Priori A, Giannicola G, Rosa M, Marceglia S, Servello D, Sassi M, Porta M (2013). Deep brain electrophysiological recordings provide clues to the pathophysiology of Tourette syndrome. Neurosci Biobehav Rev.

[CR143] Proenca CC, Gao KP, Shmelkov SV, Rafii S, Lee FS (2011). Slitrks as emerging candidate genes involved in neuropsychiatric disorders. Trends Neurosci.

[CR144] Rajagopala S, Serib S, Cavanna AE (2013). Premonitory urges and sensorimotor processing in Tourette syndrome. Behav Neurol.

[CR145] Rivlin-Etzion M, Marmor O, Heimer G, Raz A, Nini A, Bergman H (2006). Basal ganglia oscillations and pathophysiology of movement disorders. Curr Opin Neurobiol.

[CR146] Rizzo R, Curatolo P, Gulisano M, Virzi M, Arpino C, Robertson MM (2007). Disentangling the effects of Tourette syndrome and attention deficit hyperactivity disorder on cognitive and behavioral phenotypes. Brain Dev.

[CR147] Robertson MM (2000). Tourette syndrome, associated conditions and the complexities of treatment. Brain.

[CR148] Robertson MM (2008). The prevalence and epidemiology of Gilles de la Tourette syndrome. Part 1: the epidemiological and prevalence studies. J Psychosom Res.

[CR149] Robertson MM, Orth M (2006). Behavioral and affective disorders in Tourette syndrome. Adv Neurol.

[CR150] Roessner V, Becker A, Banaschewski T, Freeman RD, Rothenberger A, Tourette Syndrome International Database C (2007). Developmental psychopathology of children and adolescents with Tourette syndrome—impact of ADHD. Eur Child Adolesc Psychiatry.

[CR151] Roessner V, Plessen K, Rothenberger A (2011). European clinical guidelines for Tourette syndrome and other tic disorders. Part II: pharmacological treatment. Eur Child Adolesc Psychiatry.

[CR152] Salgado JV, Sandner G (2013). A critical overview of animal models of psychiatric disorders: challenges and perspectives. Rev Bras Psiquiatr.

[CR153] Sassi M, Porta M, Servello D (2011). Deep brain stimulation therapy for treatment-refractory Tourette’s syndrome: a review. Acta Neurochir.

[CR154] Scharf JM, Yu D, Mathews CA (2013). Genome-wide association study of Tourette’s syndrome. Mol Psychiatry.

[CR155] Schrock LE, Mink JW, Woods DW (2014). Tourette syndrome deep brain stimulation: a review and updated recommendations. Mov Disord.

[CR156] Schultz W (2004). Neural coding of basic reward terms of animal learning theory, game theory, microeconomics and behavioural ecology. Curr Opin Neurobiol.

[CR157] Servello D, Sassi M, Brambilla A, Porta M, Haq I, Foote KD, Okun MS (2009). De novo and rescue DBS leads for refractory Tourette syndrome patients with severe comorbid OCD: a multiple case report. J Neurol.

[CR158] Servello D, Zekaj E, Saleh C, Lange N, Porta M (2016). Deep brain stimulation in Gilles de la Tourette syndrome: what does the future hold? A cohort of 48 patients. Neurosurgery.

[CR159] Shibasaki H, Hallett M (2006). What is the Bereitschaftspotential?. Clin Neurophysiol.

[CR160] Shprecher DR, Gannon K, Agarwal N, Shi X, Anderson JS (2014). Elucidating the nature and mechanism of tic improvement in tourette syndrome: a pilot study. Tremor Other Hyperkinet Mov.

[CR161] Singer HS (2005). Tourette’s syndrome: from behaviour to biology. Lancet Neurol.

[CR162] Singer HS (2010). Treatment of tics and tourette syndrome. Curr Treat Options Neurol.

[CR163] Singer HS, Reiss AL, Brown JE (1993). Volumetric MRI changes in basal ganglia of children with Tourette’s syndrome. Neurology.

[CR164] Smith Y, Galvan A, Ellender TJ (2014). The thalamostriatal system in normal and diseased states. Front Syst Neurosci.

[CR165] Sowell ER, Kan E, Yoshii J (2008). Thinning of sensorimotor cortices in children with Tourette syndrome. Nat Neurosci.

[CR166] Steriade M, McCormick DA, Sejnowski TJ (1993). Thalamocortical oscillations in the sleeping and aroused brain. Science.

[CR167] Steriade M, Contreras D, Amzica F, Timofeev I (1996). Synchronization of fast (30–40 Hz) spontaneous oscillations in intrathalamic and thalamocortical networks. J Neurosci.

[CR168] Stern E, Silbersweig DA, Chee KY (2000). A functional neuroanatomy of tics in Tourette syndrome. Arch Gen Psychiatry.

[CR169] Swain JE, Scahill L, Lombroso PJ, King RA, Leckman JF (2007). Tourette syndrome and tic disorders: a decade of progress. J Am Acad Child Adolesc Psychiatry.

[CR170] Temel Y, Visser-Vandewalle V (2004). Surgery in Tourette syndrome. Mov Disord.

[CR171] Thomalla G, Siebner HR, Jonas M (2009). Structural changes in the somatosensory system correlate with tic severity in Gilles de la Tourette syndrome. Brain.

[CR172] Thomalla G, Jonas M, Baumer T (2014). Costs of control: decreased motor cortex engagement during a Go/NoGo task in Tourette’s syndrome. Brain.

[CR173] Tinaz S, Belluscio BA, Malone P, van der Veen JW, Hallett M, Horovitz SG (2014). Role of the sensorimotor cortex in Tourette syndrome using multimodal imaging. Hum Brain Mapp.

[CR174] Tinaz S, Malone P, Hallett M, Horovitz SG (2015). Role of the right dorsal anterior insula in the urge to tic in Tourette syndrome. Mov Disord.

[CR175] Tobe RH, Bansal R, Xu D, Hao X, Liu J, Sanchez J, Peterson BS (2010). Cerebellar morphology in Tourette syndrome and obsessive–compulsive disorder. Ann Neurol.

[CR176] Tykocki T, Nauman P, Koziara H, Mandat T (2013). Microlesion effect as a predictor of the effectiveness of subthalamic deep brain stimulation for Parkinson’s disease. Stereotact Funct Neurosurg.

[CR177] Uhlhaas PJ, Singer W (2006). Neural synchrony in brain disorders: relevance for cognitive dysfunctions and pathophysiology. Neuron.

[CR178] van de Griendt JM, Verdellen CW, van Dijk MK, Verbraak MJ (2013). Behavioural treatment of tics: habit reversal and exposure with response prevention. Neurosci Biobehav Rev.

[CR179] van der Salm SM, Tijssen MA, Koelman JH, van Rootselaar AF (2012). The bereitschaftspotential in jerky movement disorders. J Neurol Neurosurg Psychiatry.

[CR180] Vandewalle V, van der Linden C, Groenewegen HJ, Caemaert J (1999). Stereotactic treatment of Gilles de la Tourette syndrome by high frequency stimulation of thalamus. Lancet.

[CR181] Verdellen C, van de Griendt J, Hartmann A, Murphy T (2011). European clinical guidelines for Tourette syndrome and other tic disorders. Part III: behavioural and psychosocial interventions. Eur Child Adolesc Psychiatry.

[CR182] Volkmann J, Daniels C, Witt K (2010). Neuropsychiatric effects of subthalamic neurostimulation in Parkinson disease. Nat Rev Neurol.

[CR183] Watson TS, Dufrene B, Weaver A, Butler T, Meeks C (2005). Brief antecedent assessment and treatment of tics in the general education classroom: a preliminary investigation. Behav Modif.

[CR184] Welter ML, Mallet L, Houeto JL (2008). Internal pallidal and thalamic stimulation in patients with Tourette syndrome. Arch Neurol.

[CR185] Wichmann T, DeLong MR (1996). Functional and pathophysiological models of the basal ganglia. Curr Opin Neurobiol.

[CR186] Wong DF, Kuwabara H, Schretlen DJ (2006). Increased occupancy of dopamine receptors in human striatum during cue-elicited cocaine craving. Neuropsychopharmacology.

[CR187] Wong DF, Brasic JR, Singer HS (2008). Mechanisms of dopaminergic and serotonergic neurotransmission in Tourette syndrome: clues from an in vivo neurochemistry study with PET. Neuropsychopharmacology.

[CR188] Woods DW, Watson TS, Wolfe E, Twohig MP, Friman PC (2001). Analyzing the influence of tic-related talk on vocal and motor tics in children with Tourette’s syndrome. J Appl Behav Anal.

[CR189] Woods DW, Piacentini J, Himle MB, Chang S (2005). Premonitory Urge for Tics Scale (PUTS): initial psychometric results and examination of the premonitory urge phenomenon in youths with Tic disorders. J Dev Behav Pediatr.

[CR190] Worbe Y, Malherbe C, Hartmann A (2012). Functional immaturity of cortico-basal ganglia networks in Gilles de la Tourette syndrome. Brain.

[CR191] Worbe Y, Marrakchi-Kacem L, Lecomte S (2015). Altered structural connectivity of cortico-striato-pallido-thalamic networks in Gilles de la Tourette syndrome. Brain.

[CR192] Yu D, Mathews CA, Scharf JM (2015). Cross-disorder genome-wide analyses suggest a complex genetic relationship between Tourette’s syndrome and OCD. Am J Psychiatry.

[CR193] Zhuang P, Hallett M, Zhang X, Li J, Zhang Y, Li Y (2009). Neuronal activity in the globus pallidus internus in patients with tics. J Neurol Neurosurg Psychiatry.

[CR194] Zohar AH, Ratzoni G, Pauls DL (1992). An epidemiological study of obsessive–compulsive disorder and related disorders in Israeli adolescents. J Am Acad Child Adolesc Psychiatry.

[CR195] Zou QH, Zhu CZ, Yang Y (2008). An improved approach to detection of amplitude of low-frequency fluctuation (ALFF) for resting-state fMRI: fractional ALFF. J Neurosci Methods.

